# Extracellular matrix protein 1 regulates cell proliferation and trastuzumab resistance through activation of epidermal growth factor signaling

**DOI:** 10.1186/s13058-014-0479-6

**Published:** 2014-12-11

**Authors:** Kyung-min Lee, Keesoo Nam, Sunhwa Oh, Juyeon Lim, Young-Pil Kim, Jong Won Lee, Jong-Han Yu, Sei-Hyun Ahn, Sung-Bae Kim, Dong-Young Noh, Taehoon Lee, Incheol Shin

**Affiliations:** 1 Department of Life Science, Hanyang University, 222 Wangshimni-ro, Seoul, 133-791 Republic of Korea; 2 Department of Surgery, College of Medicine, University of Ulsan and Asan Medical Center, 88 Olympic 43-ro, Seoul, 138-736 Republic of Korea; 3 Department of Oncology, College of Medicine, University of Ulsan and Asan Medical Center, 88 Olympic 43-ro, Seoul, 138-736 Republic of Korea; 4 Cancer Research Institute, Seoul National University College of Medicine, 101 Daehak-ro, Seoul, 110-744 Republic of Korea; 5 NOVA Cell Technology, 77 Cheongam-ro, Pohang, 790-784 Republic of Korea; 6 Natural Science Institute, Hanyang University, 222 Wangshimni-ro, Seoul, 133-791 Republic of Korea

## Abstract

**Introduction:**

Extracellular matrix protein 1 (ECM1) is a secreted glycoprotein with putative functions in cell proliferation, angiogenesis and differentiation. Expression of ECM1 in several types of carcinoma suggests that it may promote tumor development. In this study, we investigated the role of ECM1 in oncogenic cell signaling in breast cancer, and potential mechanisms for its effects.

**Methods:**

In order to find out the functional role of ECM1, we used the recombinant human ECM1 and viral transduction systems which stably regulated the expression level of ECM1. We examined the effect of ECM1 on cell proliferation and cell signaling *in vitro* and *in vivo*. Moreover, tissues and sera of patients with breast cancer were used to confirm the effect of ECM1.

**Results:**

ECM1 protein was increased in trastuzumab-resistant (TR) cells, in association with trastuzumab resistance and cell proliferation. Through physical interaction with epidermal growth factor receptor (EGFR), ECM1 potentiated the phosphorylation of EGFR and extracellular signal-regulated kinase upon EGF treatment. Moreover, ECM1-induced galectin-3 cleavage through upregulation of matrix metalloproteinase 9 not only improved mucin 1 expression, but also increased EGFR and human epidermal growth factor receptor 3 protein stability as a secondary signaling.

**Conclusions:**

ECM1 has important roles in both cancer development and trastuzumab resistance in breast cancer through activation of EGFR signaling.

**Electronic supplementary material:**

The online version of this article (doi:10.1186/s13058-014-0479-6) contains supplementary material, which is available to authorized users.

## Introduction

Extracellular matrix protein 1 (ECM1) is a glycosylated protein, secreted extracellularly, that was first identified in osteogenic stromal cells [1]. The physiological function of ECM1 was first reported in keratinocytes, in which the gene was mapped to chromosome 1q21 near the epidermal differentiation complex and shown to express ECM1a and ECM1b by differentiation-dependent alternative splicing [2]. Recombinant ECM1 promotes endothelial cell proliferation and blood vessel formation, suggesting a functional role in angiogenesis [3]. Mutation in *ECM1* leads to lipoid proteinosis, an autosomal recessive skin disorder [4]. Clinically and experimentally, angiogenesis is associated with tumor progression. High levels of ECM1 expression are detected in aggressive tumorigenic cancer cell lines MDA-MB-435 and LCC15 [3], and in human carcinomas, including those of the lung, prostate, colon and breast, and especially in ductal breast carcinomas [5]. ECM1 expression is also correlated with poor prognosis [6] and metastatic potential in cancer [5],[7]. However, mechanism(s) by which ECM1 may influence tumorigenesis are unclear.

Trastuzumab (Ttzm) is a monoclonal antibody that binds to the target protein HER2 and may inhibit growth of tumor cells that overexpress HER2 [8]. The antitumoral effect of Ttzm in breast cancer may involve suppression of Akt and extracellular signal-regulated kinase (ERK) signaling and of cell cycle regulators, including cyclin D1 and p27 [9]. Ttzm is currently accepted as a principal treatment for HER2-positive breast cancer [10]. However, a significant proportion of HER2-positive tumors do not respond to or eventually escapes from Ttzm [11]. Ttzm resistance is associated with high levels of EGF signaling activity [12] and interactions of HER2 with other receptors, including HER3 and insulin-like growth factor 1 receptor [13]. In some patients, elevated p27 expression [14], loss of *PTEN* [15] and activation of phosphatidylinositol 3-kinase signaling [16] are related to Ttzm resistance. In Jimt-1 cells, mucin 4 (MUC4), by masking HER2, may disrupt binding between HER2 and Ttzm and thereby inhibit the action of Ttzm [17]. The accumulation of HER2 extracellular domain fragments in serum through shedding of HER2 is reported to induce Ttzm resistance [18]. Cellular processes, including glucose metabolism [19] and epithelial-to-mesenchymal transition (EMT) [20], may contribute as well.

In this study, we investigated the involvement of ECM1 in development of Ttzm resistance. We established Ttzm-resistant BT-474 (BT-474 TR) cells through *in vivo* xenograft systems. We compared the full spectra of proteins expressed and proteins secreted (the proteome and secretome) of BT-474 TR cells with those of control cells using two-dimensional digest (ChemDigest/Trypsin) liquid chromatography-tandem mass spectrometry (LC-MS/MS) and identified ECM1 as a Ttzm resistance biomarker protein. Our findings showed that ECM1 may influence cell proliferation and Ttzm resistance in human breast cancer cells through augmentation of EGF signaling.

## Methods

### Cell lines, antibodies, reagents and plasmids

Human breast carcinoma cell lines BT-474, MCF-7, SKBR3, MDA-MB-231, T47D, MDA-MB-468 were obtained from the American Type Culture Collection (ATCC, Manassas, VA, USA). All cells were cultured according to the recommended conditions of ATCC. Mitogen-activated protein kinase kinase (MEK) inhibitor U0126 was obtained from Calbiochem (San Diego, CA, USA). Ttzm was obtained from Roche Applied Science (Indianapolis, IN, USA). Cycloheximide was obtained from Sigma-Aldrich (St Louis, MO, USA). Recombinant ECM1 and matrix metalloproteinase 9 (MMP9) were purchased from R&D Systems (Minneapolis, MN, USA). A plasmid containing human ECM1 was made by PCR cloning from pCMV-AC-ECM1 (OriGene Technologies, Rockville, MD, USA), and cloning the gene into the pBABE-puro vector using BamHI and EcoRI restriction enzymes. The ECM1 short-hairpin RNA (shRNA) was obtained from Santa Cruz Biotechnology (Santa Cruz, CA, USA). The MMP9-Luc plasmid was kindly provided by Professor Jan Šmarda (Masaryk University, Brno, Czech Republic). Wild-type (ERK1-WT) was donated by Dr. Su-Jae Lee (Hanyang University, Seoul, Korea).

### Tumor xenografts

Four-week-old female BALB/c nude mice (ORIENT BIO, Gyeonggi-do, Korea) were implanted with 0.72-mg, 60-day release, 17β-estradiol pellets (Innovative Research, Sarasota, FL, USA). Twenty million BT-474 WT cells suspended in 200 μl of phosphate-buffered saline (PBS) were injected subcutaneously into the flank of the mice via a 22-gauge, 1.5-inch needle the next day. When the tumors reached a volume of greater than 250 mm^3^, 20 mg/kg Ttzm diluted in sterile PBS was injected into the mice by intraperitoneal injection every 3 days. The tumor volume was calculated by using the following formula: Volume (mm^3^) = Width^2^ × Length/2. We established BT-474 TR cells according to a previously described method [12]. The experiments were approved by the Hanyang University Institutional Animal Care and Use Committee, headed by Doo-Jin Park, MD, PhD, with (approval HY-IACUC-13-025). The committee strictly follows internationally recognized guidelines.

### Western blot analysis

SDS-PAGE and immunoblotting were performed according to a standard procedure. ECM1, HER3, phosphorylated ERK (p-ERK), ERK, actin, MUC1 and galectin-3 antibodies were obtained from Santa Cruz Biotechnology. c-Raf, p-c-Raf, MEK, p-MEK, Akt, p-Akt antibodies were obtained from Cell Signaling Technology (Danvers, MA, USA). Epidermal growth factor receptor (EGFR) antibody was obtained from Abcam (Cambridge, UK). HER2 antibody was obtained from Thermo Scientific (Waltham, MA, USA).

### Proliferation and viability assays

Thirty thousand cells were seeded into each well of 12-well plates. Cells were trypsinized and counted using a hemocytometer at 24, 48 and 72 hours after seeding. For 3-(4,5-dimethylthiazol-2-yl)-2,5-diphenyltetrazolium bromide (MTT) assays, cells were seeded in 96-well plates at 5 × 10^3^ cells per well, exposed to indicated antibodies and incubated for 48 or 72 hours. Cell viability was assessed by adding 20 μl of 10 mg/ml MTT (Sigma-Aldrich) to 100 μl of culture medium, and incubating for 3 hours. The medium was removed, formazan was dissolved in dimethyl sulfoxide, and the optical density was measured at 590 nm using a Multiskan EX (Thermo Scientific).

### Two-dimensional digest LCMS/MS and data analysis

The proteomes and secretomes from BT-474 TR and BT-474 WT cells were analyzed as described previously [21].

### Cell cycle analysis

Cells were harvested with 0.25% trypsin/ethylenediaminetetraacetic acid and washed once with PBS. The cells were fixed in 100% ice-cold methanol for 24 hours at −20°C. Fixed cells were incubated with 25 μg/ml propidium iodide in PBS and 1 mg/ml RNase in PBS for 30 minutes at 4°C. Cell cycle analyses were performed on a fluorescence-activated cell sorter (BD Biosciences, Franklin Lakes, NJ, USA).

### Real-time quantitative PCR

cDNA was synthesized from 5 μg of total RNA from each sample using the GeneAmp RNA PCR Core Kit (Applied Biosystems, Foster City, CA, USA). The cDNA were amplified with KAPA SYBR FAST Universal qPCR kit (Kapa Biosystems, Wilmington, MA, USA) using the following primers (Additional file 1: Table S1). The quantitative RT-PCR (RT-qPCR) experiments were performed using a Thermal Cycler Dice Real Time System TP850 (Takara Bio, Otsu, Japan).

### Immunoprecipitation

One-milligram samples of cell lysates were precleared by adding 30 μl of Protein A Sepharose (Invitrogen, Grand Island, NY, USA) for 2 hours at 4°C. After centrifugation, the supernatants were incubated with either primary antibodies or normal immunoglobulin G (IgG) overnight at 4°C. Protein A or G Sepharose (30 μl), selected according to primary antibody, was added and incubated for 3 hours at 4°C. After centrifugation, the pellet was washed three times with cell lysis buffer containing 1 mM phenylmethylsulfonyl fluoride. Immunoprecipitates were resolved by SDS-PAGE and analyzed by Western blotting.

### Fluorescence resonance energy transfer–based MMP2/9 activity assay

To detect MMP2/9 activity, peptides cleaved specifically by MMP2/9 and linked to an activatable fluorescent molecular probe (fluorescein isothiocyanate (FITC)-Gly-Pro-Leu-Gly-Val-Arg-Gly-Dabcyl (FITC-GPLG/VRG-Dabcyl)) were synthesized, based on MMP2/9-specific peptide sequences presented in previous studies [22]. Cells were incubated in serum-free medium for 24 hours, and the medium was then collected and reacted with MMP2/9 substrate peptides for 20 hours in the dark. Relative fluorescence units were determined at 480 to 620 nm using a Varioskan Flash multimode reader (Thermo Scientific).

### Dual luciferase assay

Cells seeded into 12-well plates were transfected with reporter constructs (1 μg) and pRL-CMV Renilla luciferase (5 ng) as an internal control using Lipofectamine 2000 reagent (Invitrogen). At 48 hours posttransfection, dual luciferase assays were performed according to the manufacturer’s protocol (Promega, Madison, WI, USA).

### Cell surface protein biotinylation assay

Cells at 80% to 90% confluence in 100-mm dishes were washed twice with ice-cold PBS and held for a further 15 minutes in ice-cold PBS. Biotinylation was performed by incubating cells in PBS containing 0.5 mg/ml of EZ-Link NHS-SS-Biotin (Thermo Scientific) for 30 minutes at 4°C. Biotinylated cells were washed twice with PBS and lysed. Lysates containing biotinylated proteins were precipitated with streptavidin-conjugated agarose beads (Invitrogen) and analyzed by Western blotting with the indicated antibodies. To control for nonspecific labeling of intracellular proteins, biotinylated cells were washed with 100 mM glutathione (Sigma-Aldrich) for 20 minutes at 4°C.

### Breast cancer patient serum collection

Serum samples from 20 HER2-positive breast cancer patients and accompanying data used in this study were provided and approved by the Institutional Review Board of the Asan Bio-Resource Center, Korea Biobank Network (2012–0898). Informed consent was obtained from each participant before surgery, and samples were anonymized. All samples were stored in liquid nitrogen.

### Immunocytochemistry

Cells were washed with PBS, fixed with 4% paraformaldehyde and permeabilized with 0.1% Triton X-100 for 15 minutes. Fixed samples were blocked with 3% skim milk in PBS for 1 hour, followed by incubation with primary antibody diluted in 1% skim milk in PBS for 1 hour. After being washed with PBS, the samples were treated with the anti-mouse IgG Cy3 or anti-rabbit IgG Oregon Green. For DNA staining, cells were incubated with Hoechst 33342 dye (1 μg/ml) for an additional 10 minutes. Immunofluorescence was monitored with an Olympus upright fluorescence microscope (BX50F; Olympus America, Center Valley, PA, USA).

### Statistical analysis

All data represent the results of at least three independent experiments. The data are presented as mean values ± SD. Experimental and control data were compared using Student’s *t*-test. A value of *P* < 0.05 was considered statistically significant.

### Supplementary materials and methods

Additional supplementary materials and methods can be found in Additional file 1.

## Results

### ECM1 is highly expressed in Ttzm-resistant BT-474 cells and confers Ttzm resistance

TR clones were established by transplanting HER2-overexpressing breast cancer cells into nude mice and treating the xenograft-bearing mice with Ttzm (Additional file 2: Figure S1A). The BT-474 clones selected through Ttzm treatment expressed resistance to Ttzm (Additional file 2: Figure S1B) *in vitro*, and the rate of BT-474 TR cell proliferation was increased as compared to parental BT-474 cells (Additional file 2: Figure S1C). The BT-474 TR cells displayed a smaller G_1_ fraction and increased S fraction as compared to parental BT-474 cells (Additional file 2: Figure S1D), indicating more rapid cell cycle progression in BT-474 TR cells.

**Figure 1:**
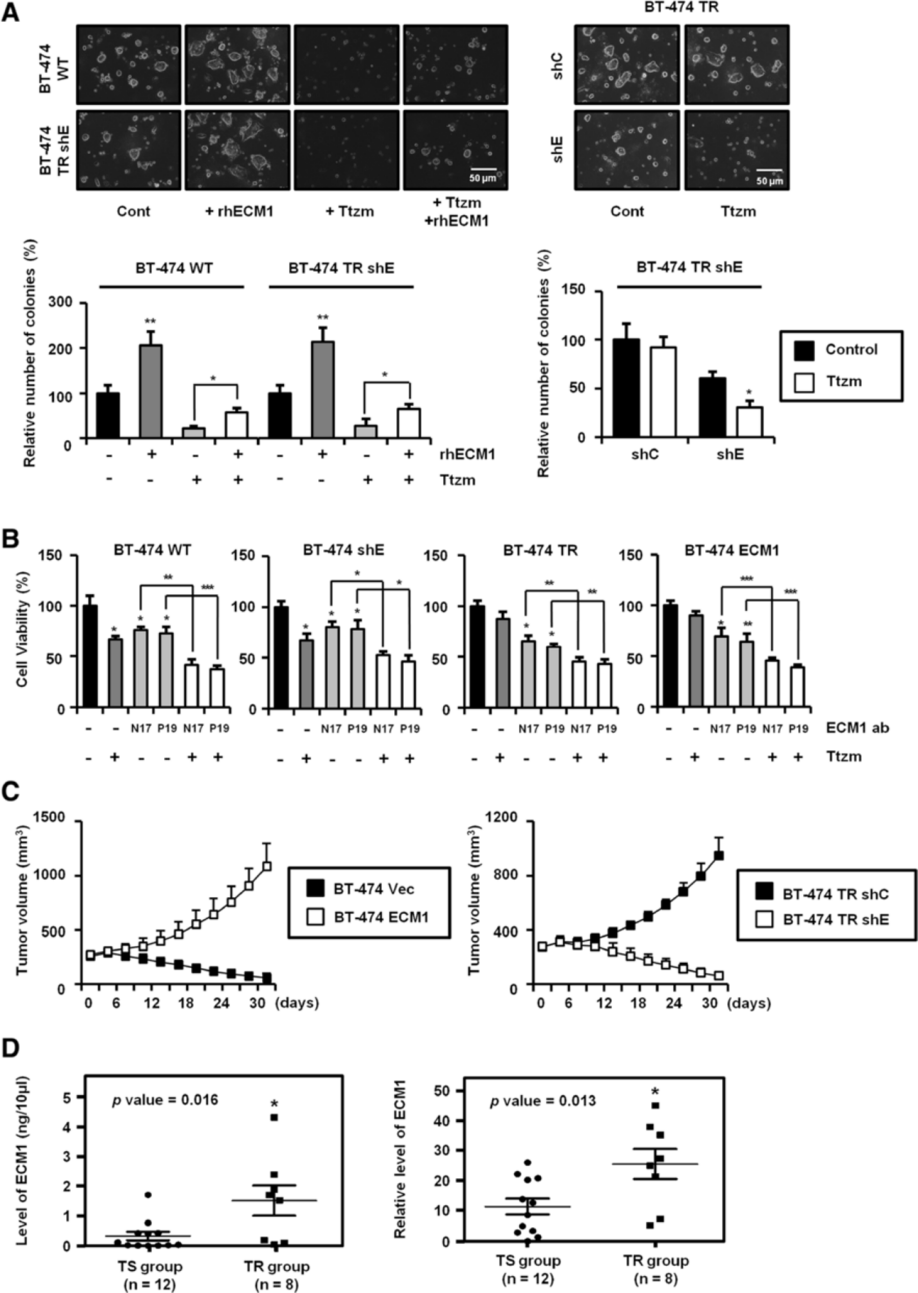
**Extracellular matrix protein 1 confers resistance toward trastuzumab. (A)** Cells were seeded with Matrigel and treated with trastuzumab (Ttzm; 20 μg/ml) and recombinant human ECM1 (rhECM1; 200 ng/ml). The number of colonies 20 μm or greater in diameter was counted at 12 days (*P < 0.05, **P < 0.005). WT, Wild type. **(B)** At 24 hours after cell seeding, each cell line was treated with anti-ECM1 antibody (5 μg/ml) and Ttzm (20 μg/ml) in fresh medium. After a further 48 hours, cell viability was analyzed using a 3-(4,5-dimethylthiazol-2-yl)-2,5-diphenyltetrazolium bromide assay (*P < 0.05, **P < 0.005, ***P < 0.0005). **(C)** BT-474 vector and ECM1 cells and BT-474 Ttzm-resistant (TR) control (Cont) short-hairpin (shRNA; shC) and ECM1 shRNA (shE) cells were passaged by subcutaneous injection into the lower flank of each mouse. When the tumor size increased up to 250 mm^3^, Ttzm at 20 mg/kg was administered to each mouse by intraperitoneal injection twice per week (*n* = 5 or 6 for each group). **(D)** Circulating levels of ECM1 in serum from TR breast cancer patients were assessed by enzyme-linked immunosorbent assay (left) and Western blot analysis and compared with (right) corresponding data for Ttzm-responsive patients (*P < 0.05).

Proteome and secretome analyses show exceptional power in identifying proteins and in tracing the complex circuitry of signals involved in tumor progression [23]. Using LC-MS/MS analysis, we identified several proteins upregulated in BT-474 TR cells as compared to BT-474 WT cells (Table 1). ECM1 expression correlates adversely with patient outcome in several types of cancer [5], and ECM1 was expressed at relatively high levels in BT-474 TR cells, at both the mRNA and protein levels (Additional file 2: Figures S1E and S1F). We evaluated the relationship of ECM1 expression to Ttzm resistance in breast cancer cells using cytotoxicity as a marker of the drug’s effectiveness. Cotreatment with recombinant human ECM1 (rhECM1) and Ttzm reduced the antiproliferative effect of Ttzm in BT-474 cells (Figure 1A, left). Conversely, ECM1 knockdown augmented Ttzm-induced cytotoxicity in BT-474 TR cells (Figure 1A, right).

**Table 1:** **Liquid chromatography-tandem mass spectrometry identification of proteins differentially secreted by BT-474 wild-type and trastuzumab-resistant BT-474 cells** ^**a**^

UniProt accession number	Protein name	Gene name	SI _WT_	SI _TR_	Log _2_(SI _TR_/SI _WT_)
Q16610	Extracellular matrix protein 1	*ECM1*	0.0762	153.8420	8.94
P27797	Calreticulin	*CALR*	8.5510	32.8933	1.3853
P07339	Cathepsin D	*CTSD*	27.7801	168.2733	2.0520
P00338	l-lactate dehydrogenase A chain	*LDHA*	55.1457	171.2111	1.0651
P04626	Receptor tyrosine protein kinase erbB-2	*ERBB2*	12.7955	33.2680	0.7694
P17931	Galectin-3	*LGALS3*	0.4470	9.4270	3.7263

^a^SI, Spectral index; TR, Trastuzumab-resistant; WT, Wild type.

In addition, direct treatment of cells with anti-ECM1 antibodies not only augmented Ttzm-induced cytotoxicity but also inhibited cell proliferation (Figure 1B). Use of two types of anti-ECM1 antibodies (N17 and P19), each specific for a different ECM1 epitope, strengthened the assumption that the antibodies had directly inhibited ECM1 activity, possibly by cross-linking the protein. The specificity of the anti-ECM1 antibody effect was further supported by the dose-dependent decrease in cell proliferation and increase in cytotoxicity in cells treated with the antibodies and Ttzm together (Additional file 2: Figure S2G). Moreover, anti-ECM1 antibodies showed higher effectiveness in BT-474 TR cells, which overexpress ECM1.

Through manipulation of ECM1 expression, we found that ECM1 may influence the suppressive effect of Ttzm on tumorigenesis in a xenograft system (Figure 1C), and sera from TR patients showed higher ECM1 levels than sera from patients with Ttzm-sensitive tumors (Figure 1D and Additional file 2: Figure S2H). These results imply that the ECM1 in patient serum may directly influence Ttzm sensitivity, consistent with the hypothesis that stable ECM1 expression confers Ttzm resistance.

### ECM1 promotes cell proliferation

**Figure 2:**
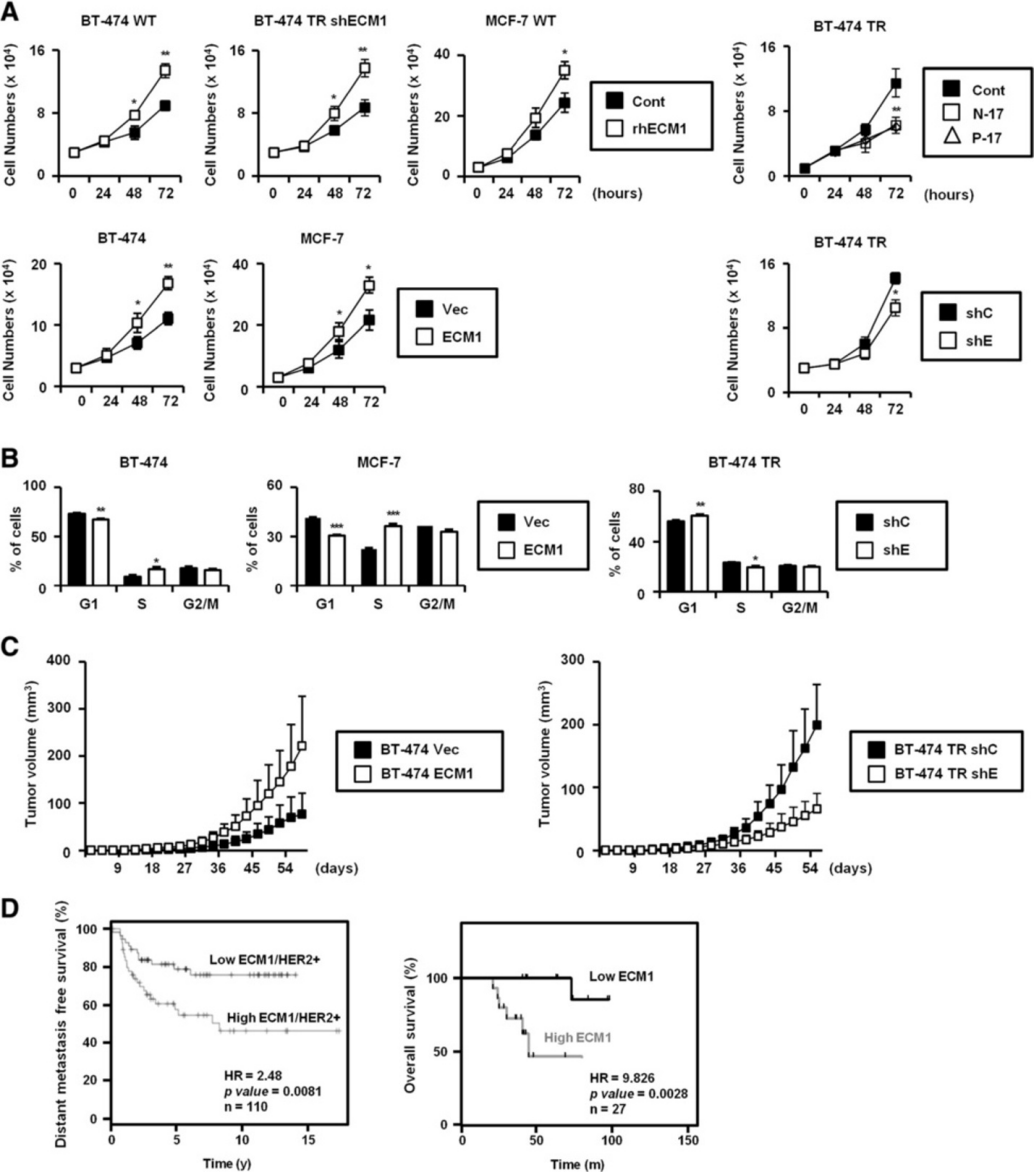
**Relationship of extracellular matrix protein 1 level to breast cancer cell proliferation. (A)** Each cell line was treated with 200 ng/ml recombinant human extracellular matrix protein 1 (rhECM1) or 5 μg/ml anti-ECM1 antibodies, and cells were counted with a hemocytometer over the course of 3 days (*P < 0.05, **P < 0.005). Cont, Control; shC, Control short-hairpin RNA; shE/shECM1, Short-hairpin extracellular matrix protein 1 RNA; TR, Trastuzumab-resistant; Vec, Vector. **(B)** Cell cycles were analyzed by flow cytometry (*P < 0.05, **P < 0.005). **(C)** Each cell line was passaged by subcutaneous injection into the lower flank of each mouse. Tumor volume was measured every 3 days (*n* = 5 or 6 for each group). **(D)** Kaplan-Meier analyses for recurrence-free survival, overall survival and distant metastasis-free survival in the HER2-positive breast cancer patients. Data were obtained from the Kaplan-Meier Plotter Breast Cancer database [24]. Overall survival data from breast cancer patients were stratified by ECM1 levels in HER2-positive patient sera (*n* = 27).

As BT-474 TR cells had a rapid proliferation ratio compared with parental BT-474 cells (Additional file 2: Figures S1C and S1D), we hypothesized that ECM1 can promote cell proliferation. To test the direct effect of ECM1 on cell proliferation, we treated BT-474 and MCF-7 cells with rhECM1. As determined by cell counting, this treatment increased the cell proliferation rate in these breast cancer cell lines (Figure 2A). To further test this effect, we overexpressed ECM1 in the BT-474 and MCF-7 cells and silenced ECM1 in BT-474 TR cells. Relative protein levels confirmed stable ECM1 expression (Additional file 3: Figure S2A), and this overexpression of ECM1 enhanced cell proliferation in the cell lines (Figure 2A, bottom). The knockdown of ECM1 by treatment with shRNA and anti-ECM1 antibodies in BT-474 TR cells reduced cell proliferation (Figure 2A, right). The anti-ECM1 antibodies also inhibited cell proliferation in other breast cancer cell lines (Additional file 3: Figure S2B). The MDA-MB-231 cell line, which overexpresses ECM1, showed especially high sensitivity to anti-ECM1 antibodies. To explore the mechanism for the cell-proliferative effect of ECM1, we analyzed the interrelationship of ECM1 expression with cell cycle progression. Cells overexpressing ECM1 showed an increased fraction of cells in S phase with a corresponding reduction of the fraction in G_1_ phase. Similarly, ECM1 knockdown led to the accumulation of cells in G_1_ phase with a decrease in the S-phase fraction (Figure 2B). Thus, ECM1 may positively regulate cell proliferation by affecting cell cycle progression. To observe the effect of ECM1 on cell proliferation *in vivo*, we tested the relationship of ECM1 expression to xenograft tumor formation in BALB/c nude mice. In BT-474 cells overexpressing ECM1, tumor formation efficiency was significantly increased, whereas tumorigenic potential of the BT-474 TR cells was significantly decreased after ECM1 silencing (Figure 2C). Moreover, the Kaplan-Meier plots [24] constructed using datasets from HER2-positive breast cancer patients demonstrated a significant association of ECM1 expression with poor outcome. Increasing ECM1 protein level predicted shorter distant metastasis-free survival in HER2-positive breast cancer patients (Figure 2D, left). Indeed, we found that high levels of ECM1 in serum were directly and inversely related to overall survival in HER2-positive breast cancer patients (Figure 2D, right). These data suggested that ECM1 overexpression in patients may contribute to bad outcomes in human breast cancer.

### ECM1 enhances EGF signaling

To investigate the mechanisms for improved proliferation by ECM1, we checked the cell signaling pathway. Because higher levels of phospho-ERK and phospho-EGFR, EGFR, and HER3 expression in BT-474 TR cells (Additional file 3: Figure S2C) and the level of ECM1 also correlated strongly with levels of phosphorylated ERK in breast tumor tissues (Additional file 3: Figure S2D), we hypothesized that ECM1 may be related to EGFR-dependent ERK activation, which can promote cell proliferation.

**Figure 3:**
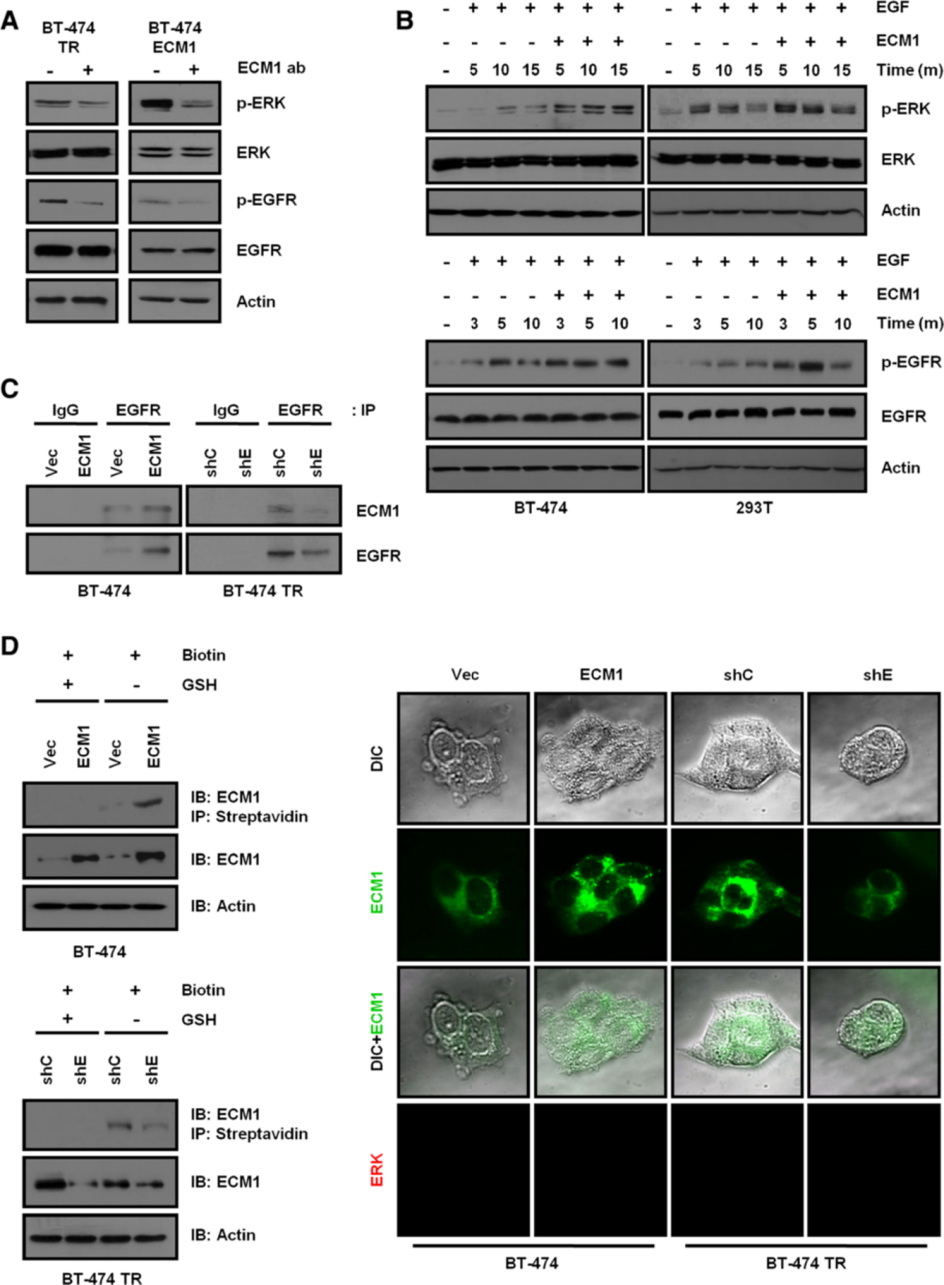
**Extracellular matrix protein 1 augments epidermal growth factor signaling. (A)** At 24 hours after seeding, BT-474 trastuzumab-resistant (TR) and BT-474 extracellular matrix protein 1 (ECM1)-expressing cells were treated with anti-ECM1 antibodies (ab; 5 μg/ml). Ten minutes later, cell lysates were analyzed on Western blots. **(B)** After serum starvation for 24 hours, cells were treated with recombinant human extracellular matrix protein 1 (rhECM1; 200 ng/ml) and epidermal growth factor (EGF; 10 ng/ml). Cell lysates were prepared at the indicated time points and analyzed on Western blots. **(C)** Total cell lysates were incubated with epidermal growth factor receptor (EGFR) antibodies overnight, and immunoprecipitates (IP) were analyzed on Western blots. IgG, Immunoglobulin G; shC, Control short-hairpin RNA; shE, Extracellular matrix protein 1; Vec, Vector. **(D)** Cells were incubated with 0.5 mg/ml EZ-Link NHS-SS-Biotin for 30 minutes at 4°C. The biotinylated proteins were precipitated by streptavidin, and the precipitates were analyzed on Western blots (IB) using ECM1 antibody (left). Cell surface labeling of ECM1 was conducted by immunostaining without permeabilization (right). Extracellular signal-regulated kinase (ERK) was used as an endogenous negative control protein. DIC, Differential Interference Contrast; GSH, Glutathione.

Because ECM1 is secreted extracellularly, we inhibited ECM1 function with anti-ECM1 antibody which neutralized the extracellular ECM1. We observed that treatment of cells with anti-ECM1 antibody reduced EGFR and ERK phosphorylation even after a treatment as brief as 10 minutes (Figure 3A). These results indicated that the activation of EGFR and ERK driven by ECM1 was initiated by the events much earlier in the signaling cascade. To confirm the effect of ECM1 on EGFR phosphorylation, we treated cells with both EGF and ECM1, and we found that this cotreatment induced higher levels of EGFR and ERK phosphorylation as compared to treatment with EGF alone (Figure 3B). However, the levels of phosphorylated EGFR and ERK proteins did not change in cells treated in serum-free medium with rhECM1 alone (Additional file 3: Figure S2E). The apparent augmentation of EGF response by ECM1 suggested that ECM1 acted at the initiation of EGFR-mediated signaling. On the basis of a report that ECM1 can physically associate with proteins bearing an EGF domain [25], we tested the interaction between ECM1 and EGF *in vitro*, but we did not observe such an association (Additional file 3: Figure S2F). However, as ECM1 is known to regulate cell signaling through association with a cell surface receptor [26], we tested the interaction between ECM1 and EGFR. By coimmunoprecipitation, ECM1 was found to interact physically with EGFR (Figure 3C), and, by immunostaining and cell surface biotinylation, ECM1 was detected at the cell surface (Figure 3D). On the basis of these results, we concluded that ECM1 may influence EGFR signaling through direct interaction with the receptor protein.

### ECM1 upregulates EGFR and HER3 expression

**Figure 4:**
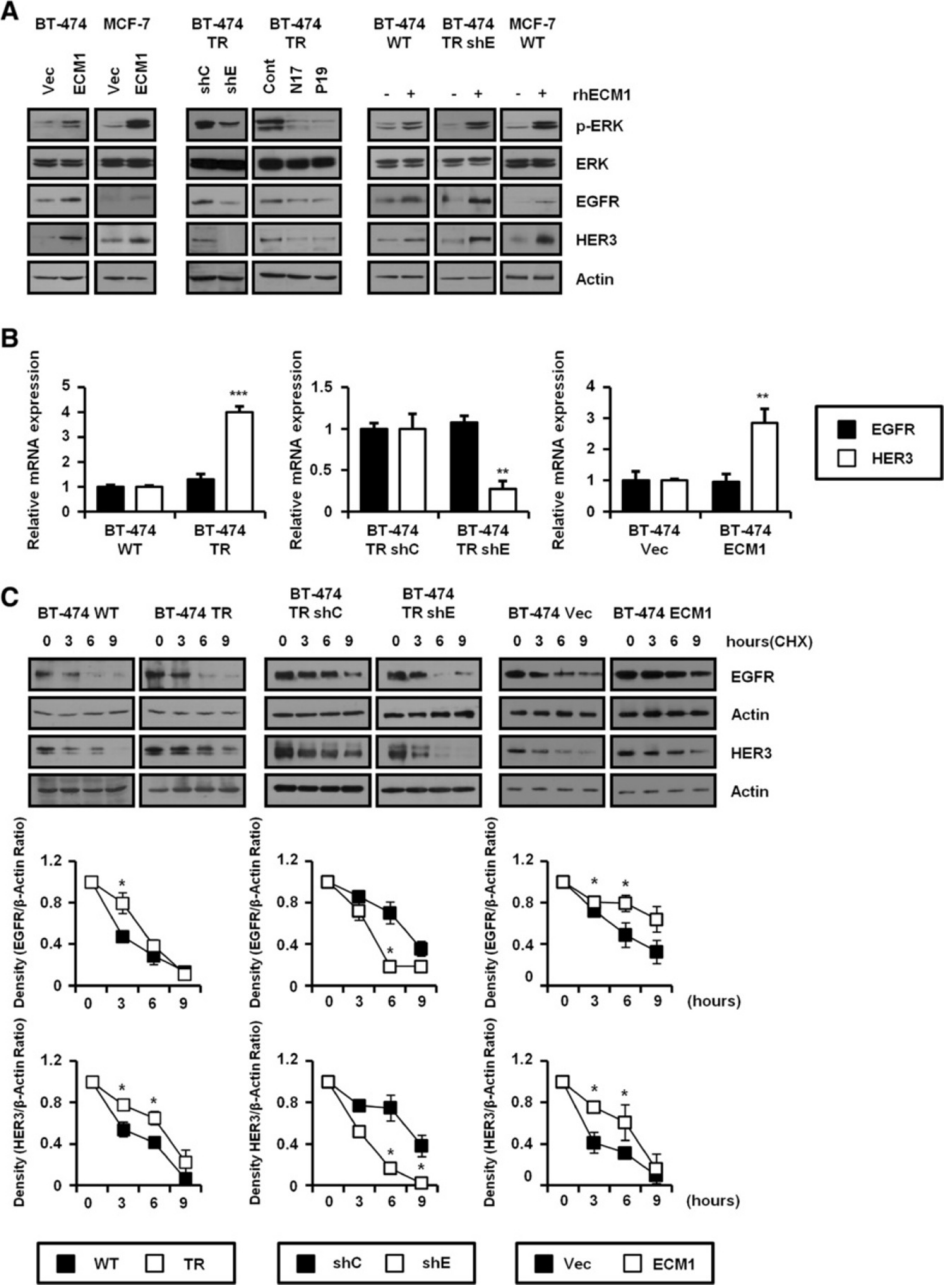
**Extracellular matrix protein 1 activates extracellular signal-regulated kinase signaling by upregulating epidermal growth factor receptor and HER3. (A)** At 24 hours after cell seeding, each cell line was treated with recombinant human extracellular matrix protein 1 (rhECM1; 200 ng/ml) or anti-ECM1 antibodies (5 μg/ml) and further incubated for 48 hours. Cells lysates were then analyzed by Western blotting. Cont, Control; ERK, Extracellular signal-regulated kinase; shC, Control short-hairpin RNA; shE, Extracellular matrix protein 1 short-hairpin RNA; TR, Trastuzumab-resistant; Vec, Vector; WT, Wild type. **(B)** Epidermal growth factor receptor (EGFR) and HER3 mRNA levels were determined by real-time PCR using primers specific for EGFR and HER3 (**P < 0.005, ***P < 0.0005). **(C)** Each cell line was treated with 100 μg/ml cycloheximide (CHX). Cell lysates were prepared at the indicated time points and analyzed on Western blots. Band intensities on the blots were quantified using 1DScan software (Scanalytics, Milwaukee, WI) and plotted versus time as the ratios of EGFR/actin and HER3/actin intensities (*P < 0.05).

Although we previously showed that ECM1 enhanced EGF-dependent activation of EGFR (Figure 3), we needed to check the effect of ECM1 on EGFR and HER3 expression because we found that EGFR and HER3 expression were increased in BT-474 TR cells compared with parental BT-474 cells (Additional file 3: Figure S2C). We first treated BT-474 and MCF-7 cells with rhECM1 for 48 hours (Figure 4A, right) and induced stable overexpression of ECM1 in the cells (Figure 4A, left). These treatments increased levels of EGFR, HER3 and phospho-ERK, proteins that were also upregulated in BT-474 TR cells. Conversely, knockdown of ECM1 in BT-474 TR cells reduced phospho-ERK levels. Consistent with these findings, treatment with anti-ECM1 antibodies (two different clones: N17 and P19) for 48 hours reduced expression of EGFR, HER3 and phospho-ERK in BT-474 TR cells (Figure 4A, middle).

Although EGFR mRNA levels did not differ significantly between BT-474 WT and BT-474 TR cells, HER3 mRNA levels were increased in BT-474 TR cells and cells overexpressing ECM1, whereas EMC1 silencing decreased the HER3 mRNA level (Figure 4B). The level of HER3 mRNA was similarly increased in ECM1-overexpressing MCF-7 cells (Additional file 4: Figure S3A). These results suggested that ECM1 augmented HER3 expression at the transcriptional level. However, ECM1 upregulation did not alter HER3 promoter activity as determined by luciferase reporter assays in BT-474 cells (Additional file 4: Figure S3B). The logical next step, then, was to determine the effect of ECM1 on EGFR and HER3 protein stabilities. In BT-474 cells, ECM1 stabilized both EGFR and HER3 proteins (Figure 4C). Taken together, these findings indicated that ECM1 influenced EGFR expression at a posttranslational level and HER3 expression at both transcriptional and posttranslational levels. The increase in cell proliferation related to ECM1 expression may involve both stabilization of EGFR and HER3 proteins and upregulation of HER3 transcription.

### EGFR and HER3 are stabilized by ECM1 through galectin-3/MUC1

**Figure 5:**
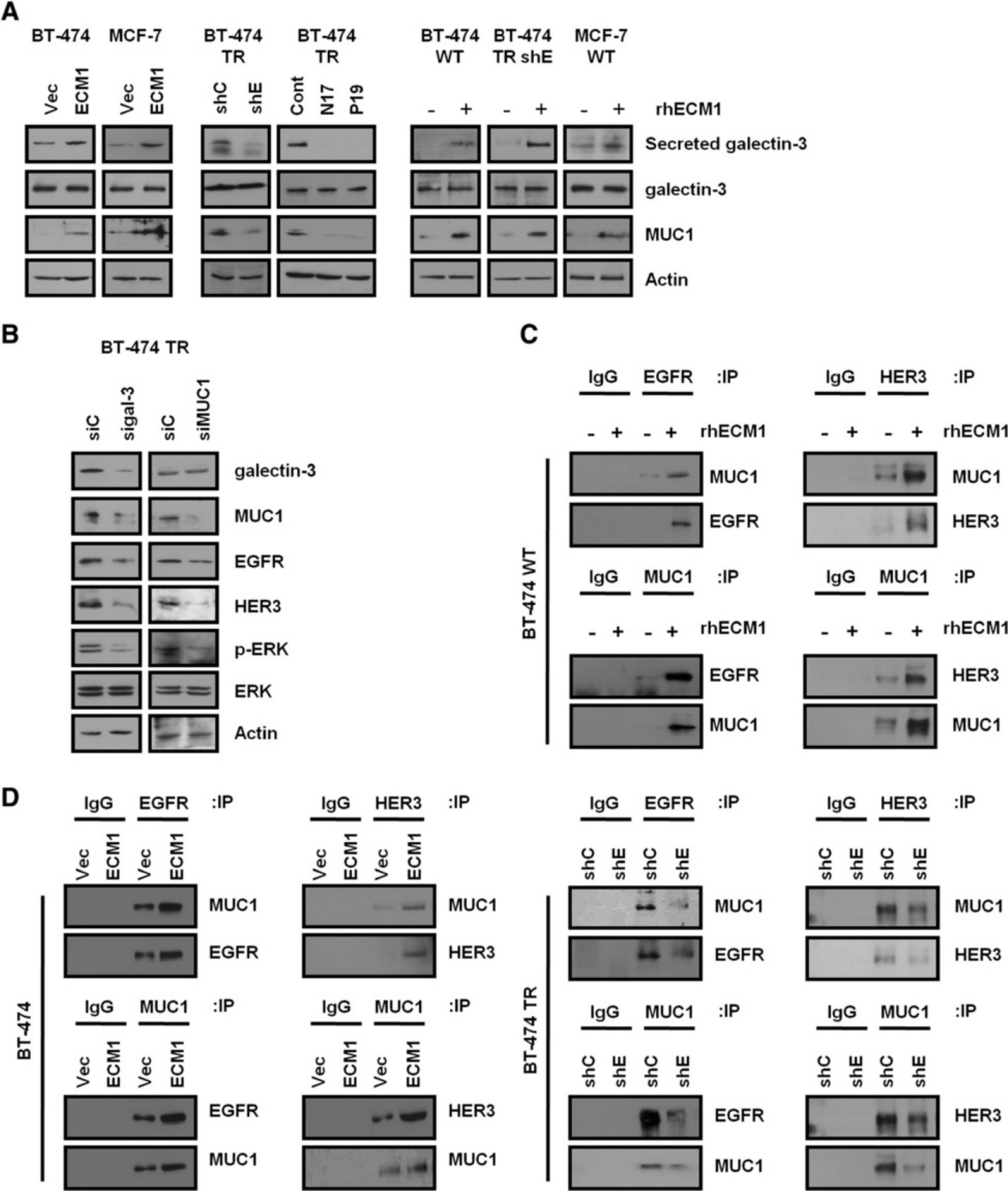
**Extracellular matrix protein 1 stabilizes epidermal growth factor receptor and HER3 proteins through galectin-3/mucin 1. (A)** Lysates from each cell line were analyzed by Western blotting. shC, Control short-hairpin RNA; shE, Extracellular matrix protein 1 short-hairpin RNA; Vec, Vector; WT, Wild type. **(B)** At 24 hours after seeding, BT-474 trastuzumab-resistant (TR) cells were transfected with each small interfering RNA (siRNA: siC, Control; sigal-3, galectin-3; siMUC1, mucin 1), incubated further for 48 hours and analyzed on Western blots. **(C)** At 24 hours after seeding, cells were treated with recombinant human extracellular matrix protein 1 (rhECM1; 200 ng/ml) and incubated further for 48 hours. Cell lysates were then incubated with mucin 1 (MUC1), epidermal growth factor receptor (EGFR) and HER3 antibodies overnight. Immunoprecipitates (IP) were analyzed on Western blots. IgG, Immunoglobulin G. **(D)** Total cell lysates were incubated with MUC1, EGFR and HER3 antibodies overnight, and immunoprecipitates were then analyzed on Western blots.

Protein stabilization, shown as an increase in protein half-life, frequently represents a physical interaction between protein molecules in the course of functional activity. The signaling and trafficking activities of EGFR, for example, involve interaction with mucin 1 (MUC1) [27]. Galectin-3, a β-galactosidase-binding secretory protein, facilitates this interaction and may thereby stabilize the other two proteins. Galectin-3 interacts with MUC1 and regulates MUC1 expression and function in cancer cells [27],[28]. Galectin-3 secretion was increased in BT-474 TR cells (Table 1). On the basis of these observations, we examined the secretion of galectin-3 and expression of MUC1 in BT-474 TR cells. Galectin-3 protein in the conditioned media and MUC1 protein content were increased in BT-474 TR cells, whereas the intracellular level of galectin-3 did not change with acquisition of Ttzm resistance (Additional file 4: Figure S3D). To test whether galectin-3 secreted into the culture medium was related to ECM1 action, we treated cultured breast cancer cells with rhECM1. Interestingly, the galectin-3 level increased in the medium, as did the level of MUC1 expression in the cells (Figure 5A, right). Similarly, ECM1 overexpression increased the levels of galectin-3 and MUC1 expression (Figure 5A, left). Conversely, ECM1 knockdown and anti-ECM1 antibody treatments reduced the levels of galectin-3 and MUC1 expression in the BT-474 TR cells (Figure 5A, middle). Although ECM1 levels influenced MUC1 protein expression, changes in ECM1 did not influence levels of MUC1 mRNA (Additional file 4: Figure S3E). These results suggested that the ECM1-related increase in galectin-3 secretion may upregulate MUC1 expression by posttranscriptional mechanisms.

To test the dependence of ECM1-related changes in EGFR and HER3 expression on galectin-3 and MUC1, we performed knockdown using small interfering RNA (Figure 5B). The knockdown of galectin-3 or MUC1 in BT-474 TR cells resulted in decreased levels of EGFR, HER3 and phospho-ERK proteins. Interestingly, galectin-3 knockdown reduced MUC1 expression, but MUC1 knockdown did not affect the galectin-3 level, indicating that galectin-3 acts upstream of MUC1 expression. MUC1 is known to activate ERK signaling through interaction with EGFR proteins [29] and to suppress EGFR degradation and facilitate EGFR trafficking [30]. To investigate interactions of EGFR with MUC1, as well as of HER3 with MUC1, that could potentially stabilize EGFR or HER3, we coimmunoprecipitated these proteins in BT-474 cells. Interactions between MUC1 and EGFR/HER3 were increased in BT-474 TR cells (Additional file 4: Figure S3F). Likewise, rhECM1 treatment and ECM1 overexpression enhanced interactions between MUC1 and EGFR/HER3 in BT-474 cells (Figures 5C and 5D, left), whereas knockdown of ECM1 in BT-474 TR cells reduced these interactions (Figure 5D, right). Colocalizations of EGFR-MUC1 and of HER3-MUC1 were also increased in TR cells by ECM1 expression (Additional file 4: Figure S3G). Thus, ECM1 may stabilize EGFR and HER3 by promoting interactions of these proteins with galectin-3 and MUC1.

### ECM1 promotes MMP9 expression

**Figure 6:**
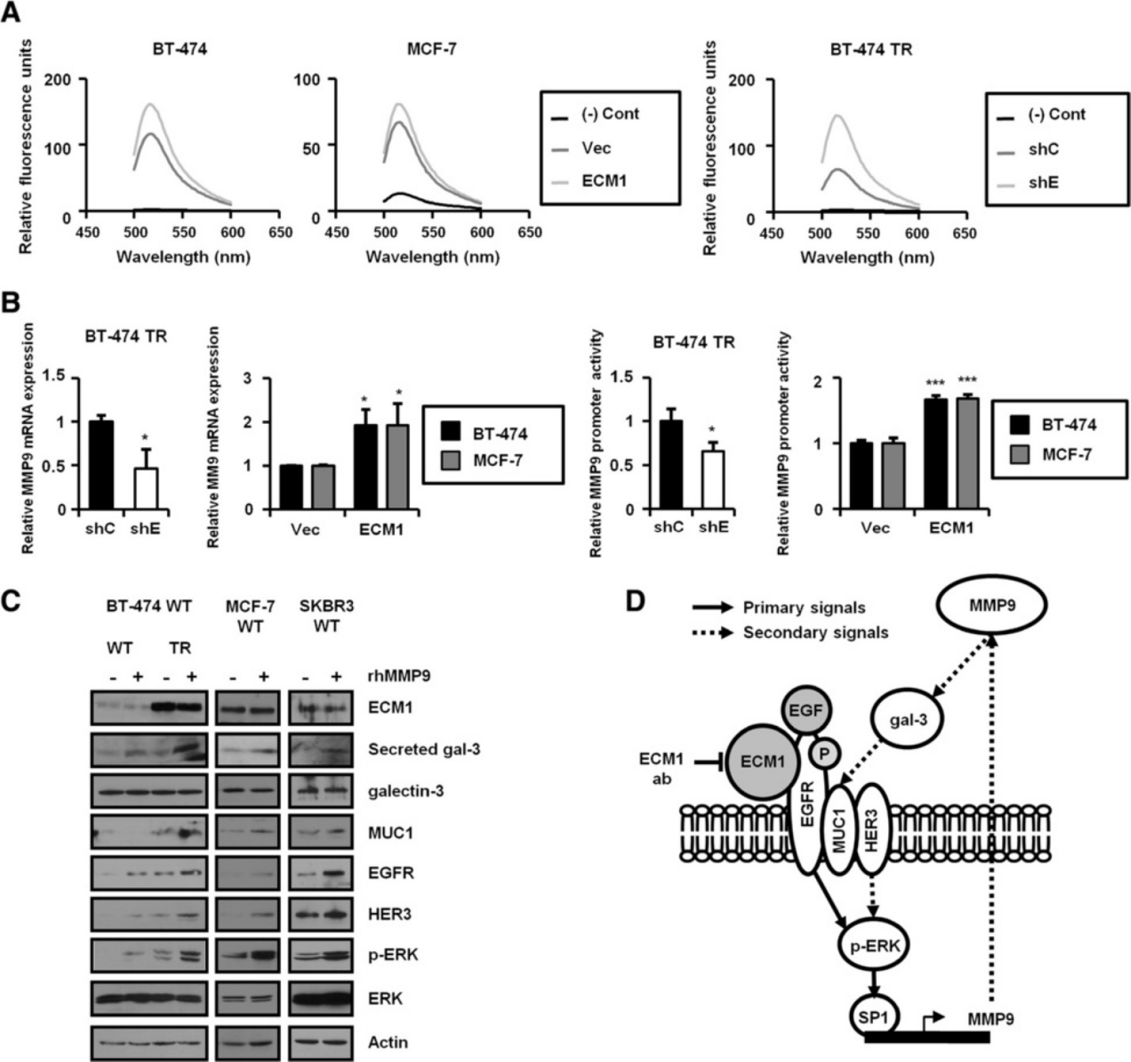
**Extracellular matrix protein 1 induces matrix metalloproteinase 9 transcription. (A)** At 24 hours after seeding, cells were incubated with serum-free medium for a further 24 hours. Supernatant medium from each cell line was reacted with matrix metalloproteinase 9 (MMP9) substrate, and relative fluorescence units were determined at 480 to 620 nm. Cont, Control; shC, Control short-hairpin RNA; shE, Extracellular matrix protein 1 short-hairpin RNA; TR, Trastuzumab-resistant; Vec, Vector. **(B)** MMP9 mRNA levels were determined by real-time PCR using primers specific for MMP9 (*P < 0.05). Each cell line was transfected with an MMP9 promoter luciferase reporter construct. After 48 hours, cells were harvested, and the lysates were analyzed by dual-luciferase assay (*P < 0.05, ***P < 0.0005). **(C)** At 24 hours after seeding, each cell line was treated with recombinant human MMP9 (rhMMP9; 20 ng/ml), incubated further for 48 hours, and lysates from each cell were analyzed by Western blotting. EGFR, Epidermal growth factor receptor; ERK, Extracellular signal-regulated kinase; MUC1, Mucin 1; WT, Wild type. **(D)** Schematic model showing the role of extracellular matrix protein 1 (ECM1) in cell signaling. EGF, Epidermal growth factor.

The ECM1-dependent increase in galectin-3 secretion by cultured cells implied the intervention of a factor upstream, such as MMP2/9, which acts on galectin-3 as a substrate in developmental processes [31]. Mutation at the MMP9 cleavage site in galectin-3 reduces extracellular galectin-3 and also suppresses tumorigenicity of breast cancer cells [32]. Using zymography and a fluorescence resonance energy transfer (FRET)-based assay for MMP2 and MMP9 activities, we measured higher MMP9 activity in BT-474 TR cells than in BT-474 WT cells (Additional file 5: Figures S4A and S4B). Knockdown of ECM1 reduced MMP9 activity in BT-474 TR cells, and ECM1 overexpression increased MMP9 activity in BT-474 and MCF-7 cells (Figure 6A). Although our FRET assays employed an MMP substrate susceptible to both MMP2 and MMP9, we found that modulation of ECM1 resulted in increased MMP9 activity, whereas MMP2 activity remained constant as determined by gelatin zymography assay (Additional file 5: Figure S4B). In a cell-free system, changes in ECM1 expression did not influence MMP9 activity (Additional file 5: Figure S4C). In BT-474 TR cells, however, the MMP9 mRNA level was higher than in control cells (Additional file 5: Figure S4D), and, in both BT-474 and MCF-7 cells, changes in ECM1 expression correlated with changes in MMP9 transcript levels (Figure 6B, left). In accord with previous results, MMP9 promoter activity increased following the acquisition of the TR phenotype and increases in ECM1 expression in BT-474 and MCF-7 cells (Figure 6B, right). These results may mean that ECM1 regulates MMP9 expression at the transcriptional level. The transcription factor Sp1 is reported to bind at the MMP9 promoter upon ERK activation [33]; hence, we speculated that ERK might influence MMP9 transcription through Sp1 activation. Seeking to link ECM1 activity with MMP9 transcription, we investigated the regulatory signaling of MMP9 expression. In BT-474 cells, we found that ERK overexpression induced MMP9 transcription (Additional file 5: Figure S4E) and that treatment with U0126, a specific inhibitor of MEK, reduced MMP9 transcription (Additional file 5: Figure S4F). These results may indicate that ERK activates Sp1, which in turn regulates MMP9 transcription. To further confirm MMP9 as a mediator of ECM1-dependent effects, we treated breast cancer cells with recombinant human MMP9 (rhMMP9). The results of this treatment included higher levels of galectin-3 in supernatant media, as well as higher levels of MUC1, EGFR and HER3 proteins. Interestingly, levels of phosphorylated ERK also increased (Figure 6C), and Ttzm-treated BT-474 cells were rescued from cytotoxicity by the rhMMP9 treatment (Additional file 5: Figure S4G). These findings suggested that ECM1 may regulate cellular events through modulation of MMP9 transcription.

## Discussion

The present study demonstrates that ECM1 may regulate both cancer cell proliferation and Ttzm resistance through interaction with EGF signaling. ECM1 was shown to associate directly with EGFR, and EGF-dependent EGFR and ERK activation followed thereafter. EGF was in turn shown to activate ERK signaling at the level of transcription, through activation of the transcription factor Sp1 and MMP9 expression proceeded downstream of these events. Galectin-3 cleavage, dependent on MMP9 upregulation, facilitated formation of a galectin-3/MUC1/EGFR complex. This cascade of signaling events led to EGFR and HER3 protein stabilization, as well as to ERK activation in breast cancer cells (schematically illustrated in Figure 6D).

ECM1-dependent upregulation of the EGF response could drive the acquisition of Ttzm resistance, as previously suggested [12]. ECM1 is known to interact with the EGF domain on other proteins, such as perlecan [25], and perlecan may induce heparin-binding growth factor responses, including the fibroblast growth factor 2 (FGF2) response, and thereby promote tumor growth [34]. Indeed, ECM1 and perlecan interact during development and in pathological events, including tumor progression. In testing the effect of ECM1 on FGF2-mediated responses (Additional file 3: Figure S2G), we found no further enhancement of FGF2 response in cells cotreated with both proteins as compared to FGF2 alone. However, although ECM1 showed no interaction with FGF2 signaling, ECM1 has been shown to associate with various other cell surface receptors [26], such as the interleukin 2 receptor. Moreover, various proteins that bind with EGFR also regulate the activation and trafficking of EGFR [30],[35]. It was recently reported that epidermal growth factor-containing fibulin-like extracellular matrix protein 1 (EFEMP1) binds to EGFR and attenuates EGFR signaling [36]. ECM1 may interrupt binding of inhibitory protein like EFEMP1. In the present study, we demonstrated that ECM1 may interact physically with EGFR and enhance EGF signaling. Interactions between ECM1 and extracellular matrix components such as laminin, collagen and fibronectin may enhance binding of these components [37]. We did not focus on these interactions, however; instead, we investigated the ECM1-regulated signaling that occurred through association with EGFR in a brief interaction with the cell.

In BT-474 TR cells, which overexpress ECM1, we showed that expression of EGFR and HER3 were increased as compared with parental BT-474 cells. An increase in expression or activation of EGFR proteins is correlated with Ttzm resistance, and Ttzm resistance has been shown to depend on EGFR expression [12]. Also, increased interaction between HER2 and HER3 promotes acquisition of Ttzm resistance [13], and HER3 upregulation may compensate for inhibition of the HER2 tyrosine kinase [38]. On the basis of our data (Figure 2D), we found that patients with highly positive ECM1/HER2 tumors had relatively poor outcomes, consistent with the idea that ECM1 may interact with EGFR family receptors that interact with HER2 to attenuate therapeutic action by Ttzm.

Also noteworthy is that galectin-3/MUC1 interactions influenced the stability of EGFR and HER3 proteins. Interaction of galectin-3 and MUC1 regulates the expression and function of MUC1 [27],[28], and galectin-3 facilitates an interaction between MUC1 and EGFR [27] that promotes EGFR activation. Galectin-3 is reported to regulate intracellular trafficking of EGFR in keratinocytes and to promote cell migration [39]. MUC1 also regulates EGFR trafficking and may inhibit EGFR degradation [30]. Accordingly, treatment of cancer cells with anti-MUC1 antibody inhibits EGFR signaling [40]. MUC1 activity is also known to contribute to Ttzm resistance [41], and a high circulating level of galectin-3 in patient serum is related to tumor progression [42].

In the present study, our secretome analysis revealed increased secretion of galectin-3 into the culture medium by TR cells (Table 1). We showed that ECM1 expression promoted EGFR and HER3 stabilities by posttranslational activities involving MUC1 and that ECM1 regulated HER3 at the mRNA level. We also monitored microRNA-205 (miR-205) expression in ECM1-expressing cells (Additional file 4: Figure S3C). miR-205 is known to inhibit HER3 expression through interaction with HER3 mRNA [43]. However, we found no evidence that ECM1 affected miR-205 levels. In addition, it seems plausible, based on our findings, that the unfavorable prognosis associated with highly MUC1/HER2-positive breast tumors (data not shown; see [24]) may be attributable to ECM1-mediated upregulation of MUC1.

Circulating serum galectin-3 may increase through MMP9-dependent cleavage of membrane-bound galectin-3 [32], and this cleavage of galectin-3 by MMP9 promotes breast cancer angiogenesis and progression [44]. Although ECM1 is reported to inhibit MMP9 activity [45], we did not observe this effect of ECM1 (Additional file 5: Figure S4C). Having shown that MMP9 mediated ECM1-dependent cellular events (Figure 6), we investigated the mechanism for ECM1-regulated MMP9 transcription. Indeed, the correlation of ECM1 expression with MMP9 levels was shown in a previous study [46], and, consistent with our results, MMP9 has been demonstrated to regulate cell proliferation through EGFR-mediated activation of ERK in Schwann cells [47]. In addition, MMP9 acts as a sheddase of HER2 [48], and an increase in circulating HER2 through shedding may be an essential factor in development of resistance to chemotherapy, including Ttzm resistance, in advanced breast cancer [49]. In BT-474 TR cells in culture, we detected higher extracellular HER2 levels than in the parent line (Table 1), and we observed that rhMMP9 augmented this effect (data not shown). Although we suggest that the main mechanism by which ECM1 regulates Ttzm resistance is through an increase of EGFR/HER3 by the activities of MMP9, we cannot rule out the possibility that upregulation of MMP9 expression by ECM1 increases circulating HER2 levels and that this source of HER2 contributes to Ttzm resistance.

In addition, our finding that ECM1 upregulates MUC1 may inform future research into the signaling that initiates and maintains malignant phenotype. MUC1 blocks degradation and nuclear accumulation of β-catenin [50], a protein with multiple regulatory functions that thus is influential in tumor development, and MUC1 regulates gene transcription by forming a complex with nuclear factor κB [51]. Moreover, MUC1 promotes a metastatic phenotype of cancer cells by inducing EMT and activating the ZEB1/miR-200c regulatory loop [52]. We suggest that ECM1 may influence the EMT, and thus metastatic potential, through regulation of MUC1 expression, in accordance with reported correlations of ECM1 expression with metastasis in laryngeal carcinoma [53] and cholangiocarcinoma [46].

## Conclusion

We found that ECM1 increased EGF-mediated signaling through interaction with EGFR and subsequent activation of the ERK pathway. Our findings are clinically relevant in identifying ECM1 as an unfavorable prognostic marker in HER2-positive breast cancer.

## Additional files

## Electronic supplementary material

Additional file 1: Table S1.: List of primers used in the study. Supplementary materials and methods. (PDF 71 KB)

Additional file 2: Figure S1.: ECM1 is over-expressed in Ttzm-resistant BT-474 cells. **(A)** 17β-estradiol pellet was injected to 4-week-old BABL/c nude mice and BT-474 WT cells were passaged in the mice by subcutaneous injection of 2 × 10^7^ cells into the lower flank of the mice. When the tumor size grew to 250 mm^3^, 20 mg/kg Ttzm was injected into the mice by intraperitoneal injection twice per week. NS-1 and NS-2, as control groups, responded to Ttzm completely. **(B)** 5 × 10^4^ cells were plated on soft agar and Matrigel (Additional file 1: Supplementary materials and methods). In Matrigel, Ttzm (20 μg/ml) was treated every 3 days. The number of colonies (20 μm diameter) was counted at 12 days. The number of colonies is quantified in right panels. Error bars represent mean ± SD of triplicate experiments (*P < 0.05, **P < 0.005). **(C)** Cells were counted with a hemocytometer over 3 days (*P < 0.05, **P < 0.005). **(D)** Cell cycles in BT-474 WT and BT-474 TR cells were analyzed using flow cytometry (*P < 0.05, **P < 0.005). **(E)** mRNAs were analyzed by RT-PCR using primers specific for ECM1 and GAPDH (Additional file 1: Supplementary materials and methods). Secreted ECM1 was obtained from Trichloroacetic acid-precipitated cell supernatant medium. Each cell lysate was analyzed by Western blotting using ECM1- and actin-specific antibodies. **(F)** ECM1 mRNA levels were determined by real-time PCR using primers specific for ECM1 (***P < 0.0005). **(G)** At 24 hours after cell seeding, each cell line was treated with anti-ECM1 antibody (5 μg/ml) and Ttzm (20 μg/ml) in fresh medium. After a further 48 hours, cell viability was analyzed using an MTT assay (*P < 0.05, **P < 0.005, ***P < 0.0005). **(H)** Levels of ECM1 in serum from Ttzm-resistant breast cancer patients were assessed Western blot analysis, and compared with corresponding data for Ttzm-responsive patients. (PDF 313 KB)

Additional file 3: Figure S2.: Functional role of ECM1 in cancer cell proliferation. **(A)** Cells lysates were analyzed by Western blotting with the indicated antibodies. **(B)** Each cell line was treated with each anti-ECM1 antibody (see Methods) at 5 μg/ml. After a further 48 hours, cell viability was analyzed using an MTT assay (*P < 0.05, **P < 0.005, ***P < 0.0005). **(C)** Cell lysates were analyzed by Western blotting using indicated antibodies. Anti-actin antibody was applied as a loading control. **(D)** Western blot analysis shows levels of p-ERK and ECM1 proteins in primary tumor lysates from breast cancer patients (*n* = 17). The positive relationship between p-ERK and ECM1 expression levels is indicated (*R* ^2^ = 0.6131). **(E)** After serum starvation for 24 hours, cells were treated with rhECM1 (200 ng/ml) for 10 minutes. Cell lysates were analyzed by Western blotting using indicated antibodies. **(F)** A mixture containing rhECM1 (500 ng) and biotin-EGF (500 ng) was incubated with streptavidin-agarose beads overnight and the immunoprecipitates were analyzed on Western blots. **(G)** At 24 hours after cell seeding, BT-474 WT cells were treated with rhECM1 (200 ng/ml) and FGF2 (10 ng/ml). The cell lysates were obtained at the indicated time points and subjected to Western blot analysis with indicated antibodies. (PDF 353 KB)

Additional file 4: Figure S3.: ECM1-dependent induction of EGFR/HER3 is mediated by MUC1. **(A)** EGFR and HER3 mRNA levels were determined by real-time PCR using primers specific for EGFR and HER3 (**P < 0.005). **(B)** Each cell was transfected with HER3 promoter luciferase reporter constructs, harvested after 48 h and analyzed by dual-luciferase assay. **(C)** Expression of miR-200c was assessed by RT-qPCR with a universal reverse primer and forward primers specific for miR-200c using a TaqMan microRNA assay kit (*P < 0.05) (Additional file 1: Supplementary materials and methods). **(D)** Cell lysates were analyzed by Western blotting using the indicated antibodies. **(E)** MUC1 mRNA levels were determined by real-time PCR using primers specific for MUC1 (*P < 0.05). **(F)** Cell lysates were incubated with MUC1, EGFR and HER3 antibodies overnight. Immunoprecipitates were analyzed on Western blots. **(G)** Colocalizations of MUC1 and EGFR/HER3 were monitored by immunostaining. Each cell was fixed and stained with indicated antibodies and Hoechst dye for nuclear staining. (PDF 294 KB)

Additional file 5: Figure S4.: ERK-dependent regulation of MMP9 transcription by ECM1. **(A)** Supernatant medium from each cell line was reacted with MMP9 substrate and relative fluorescence units were determined at 480 to 620 nm. **(B)** Conditioned media from each cell were collected, and gelatin zymography was performed. Arrows indicate MMP2 and MMP9. Each bar graph represents the quantified intensity of indicated cells, as assessed by gelatin zymography (*P < 0.05, **P < 0.005) (Additional file 1: Supplementary materials and methods). **(C)** Media containing rhMMP9 and rhECM1 were reacted with MMP9 substrate. Relative fluorescence units were determined at 480 to 620 nm. **(D)** MMP9 mRNA levels were determined by real-time PCR using primers specific for MMP9 (*P < 0.05). Each cell line was transfected with an MMP9 promoter luciferase reporter construct. After 48 h, cells were harvested and the lysates were analyzed by dual-luciferase assay (**P < 0.005). **(E)** and **(F)** Each cell was transfected with ERK1-WT constructs (E) and treated with U0126 (F). MMP9 mRNA levels were determined by real-time PCR using primers specific for MMP9 and MMP9 promoter activity was analyzed by dual-luciferase assay (*P < 0.05, **P < 0.005). **(G)** At 24 hours after cell seeding, each cell line was treated with rhMMP9 (10, 20 ng/ml) and Ttzm (20 μg/ml) and incubated further for 48 hours. Cell viability was then analyzed using an MTT assay (**P < 0.005). (PDF 150 KB)

Below are the links to the authors’ original submitted files for images.

Authors’ original file for figure 1

Authors’ original file for figure 2

Authors’ original file for figure 3

Authors’ original file for figure 4

Authors’ original file for figure 5

Authors’ original file for figure 6

## Abbreviations

ECM1: Extracellular matrix protein 1
EFEMP1: Epidermal growth factor-containing fibulin-like extracellular matrix protein 1
EGF: Epidermal growth factor
EGFR: Epidermal growth factor receptor
EMT: Epithelial-to-mesenchymal transition
ERK: Extracellular signal-regulated kinase
FGF2: Fibroblasts growth factor 2
FITC: Fluorescein isothiocyanate
FRET: Fluorescence resonance energy transfer
HER: Human epidermal growth factor receptor
IgG: Immunoglobulin G
LC-MS/MS: Liquid chromatography-tandem mass spectrometry
MEK: Mitogen-activated protein kinase kinase
MMP: Matrix metalloproteinase
MTT: 3-(4,5-dimethylthiazol-2-yl)-2,5-diphenyltetrazolium bromide
MUC: Mucin
PBS: Phosphate-buffered saline
rhECM1: Recombinant human extracellular matrix protein 1
shRNA: Short-hairpin RNA
siRNA: Small interfering RNA
TR: Trastuzumab-resistant
Ttzm: Trastuzumab
WT: Wild type

## References

[1] Mathieu E, Meheus L, Raymackers J, Merregaert J (1994). Characterization of the osteogenic stromal cell line MN7: identification of secreted MN7 proteins using two-dimensional polyacrylamide gel electrophoresis, Western blotting, and microsequencing. J Bone Miner Res.

[2] Smits P, Poumay Y, Karperien M, Tylzanowski P, Wauters J, Huylebroeck D, Ponec M, Merregaert J (2000). Differentiation-dependent alternative splicing and expression of the extracellular matrix protein 1 gene in human keratinocytes. J Invest Dermatol.

[3] Han Z, Ni J, Smits P, Underhill CB, Xie B, Chen Y, Liu N, Tylzanowski P, Parmelee D, Feng P, Ding I, Gao F, Gentz R, Huylebroeck D, Merregaert J, Zhang L (2001). Extracellular matrix protein 1 (ECM1) has angiogenic properties and is expressed by breast tumor cells. FASEB J.

[4] Hamada T, McLean WH, Ramsay M, Ashton GH, Nanda A, Jenkins T, Edelstein I, South AP, Bleck O, Wessagowit V, Mallipeddi R, Orchard GE, Wan H, Dopping-Hepenstal PJ, Mellerio JE, Whittock NV, Munro CS, van Steensel MA, Steijlen PM, Ni J, Zhang L, Hashimoto T, Eady RAJ, McGrath JA (2002). Lipoid proteinosis maps to 1q21 and is caused by mutations in the extracellular matrix protein 1 gene (*ECM1*). Hum Mol Genet.

[5] Wang L, Yu J, Ni J, Xu XM, Wang J, Ning H, Pei XF, Chen J, Yang S, Underhill CB, Liu L, Liekens J, Merregaert J, Zhang L (2003). Extracellular matrix protein 1 (ECM1) is over-expressed in malignant epithelial tumors. Cancer Lett.

[6] Lal G, Hashimi S, Smith BJ, Lynch CF, Zhang L, Robinson RA, Weigel RJ (2009). Extracellular matrix 1 (*ECM1*) expression is a novel prognostic marker for poor long-term survival in breast cancer: a hospital-based cohort study in Iowa. Ann Surg Oncol.

[7] Wu QW (2012). She HQ, Liang J, Huang YF, Yang QM, Yang QL, Zhang ZM: **Expression and clinical significance of extracellular matrix protein 1 and vascular endothelial growth factor-C in lymphatic metastasis of human breast cancer**. BMC Cancer.

[8] Carter P, Presta L, Gorman CM, Ridgway JB, Henner D, Wong WL, Rowland AM, Kotts C, Carver ME, Shepard HM (1992). Humanization of an anti-p185HER2 antibody for human cancer therapy. Proc Natl Acad Sci U S A.

[9] Yakes FM, Chinratanalab W, Ritter CA, King W, Seelig S, Arteaga CL (2002). Herceptin-induced inhibition of phosphatidylinositol-3 kinase and Akt Is required for antibody-mediated effects on p27, cyclin D1, and antitumor action. Cancer Res.

[10] Goldenberg MM (1999). Trastuzumab, a recombinant DNA-derived humanized monoclonal antibody, a novel agent for the treatment of metastatic breast cancer. Clin Therapeut.

[11] Slamon DJ, Leyland-Jones B, Shak S, Fuchs H, Paton V, Bajamonde A, Fleming T, Eiermann W, Wolter J, Pegram M, Baselga J, Norton L (2001). Use of chemotherapy plus a monoclonal antibody against HER2 for metastatic breast cancer that overexpresses HER2. N Engl J Med.

[12] Ritter CA, Perez-Torres M, Rinehart C, Guix M, Dugger T, Engelman JA, Arteaga CL (2007). Human breast cancer cells selected for resistance to trastuzumab *in vivo*overexpress epidermal growth factor receptor and ErbB ligands and remain dependent on the ErbB receptor network. Clin Cancer Res.

[13] Huang X, Gao L, Wang S, McManaman JL, Thor AD, Yang X, Esteva FJ, Liu B (2010). Heterotrimerization of the growth factor receptors erbB2, erbB3, and insulin-like growth factor-I receptor in breast cancer cells resistant to herceptin. Cancer Res.

[14] Nahta R, Takahashi T, Ueno NT, Hung MC, Esteva FJ (2004). p27^kip1^down-regulation is associated with trastuzumab resistance in breast cancer cells. Cancer Res.

[15] Nagata Y, Lan KH, Zhou X, Tan M, Esteva FJ, Sahin AA, Klos KS, Li P, Monia BP, Nguyen NT, Hortobagyi GN, Hung MC, Yu D (2004). PTEN activation contributes to tumor inhibition by trastuzumab, and loss of PTEN predicts trastuzumab resistance in patients. Cancer Cell.

[16] Berns K, Horlings HM, Hennessy BT, Madiredjo M, Hijmans EM, Beelen K, Linn SC, Gonzalez-Angulo AM, Stemke-Hale K, Hauptmann M, Beijersbergen RL, Mills GB, van de Vijver MJ, Bernards R (2007). A functional genetic approach identifies the PI3K pathway as a major determinant of trastuzumab resistance in breast cancer. Cancer Cell.

[17] Nagy P, Friedlander E, Tanner M, Kapanen AI, Carraway KL, Isola J, Jovin TM (2005). Decreased accessibility and lack of activation of ErbB2 in JIMT-1, a herceptin-resistant, MUC4-expressing breast cancer cell line. Cancer Res.

[18] Todeschini P, Cocco E, Bellone S, Varughese J, Lin K, Carrara L, Guzzo F, Buza N, Hui P, Silasi DA, Ratner E, Azodi M, Schwartz PE, Rutherford TJ, Pecorelli S, Santin AD (2011). Her2/neu extracellular domain shedding in uterine serous carcinoma: implications for immunotherapy with trastuzumab. Br J Cancer.

[19] Zhao Y, Liu H, Liu Z, Ding Y, Ledoux SP, Wilson GL, Voellmy R, Lin Y, Lin W, Nahta R, Liu B, Fodstad O, Chen J, Wu Y, Price JE, Tan M (2011). Overcoming trastuzumab resistance in breast cancer by targeting dysregulated glucose metabolism. Cancer Res.

[20] Oliveras-Ferraros C, Corominas-Faja B, Cufi S, Vazquez-Martin A, Martin-Castillo B, Iglesias JM, López-Bonet E, Martin ÁG, Menendez JA (2012). Epithelial-to-mesenchymal transition (EMT) confers primary resistance to trastuzumab (Herceptin). Cell Cycle.

[21] Park S, Lee KM, Ju JH, Kim J, Noh DY, Lee T, Shin I (2010). Protein expression profiling of primary mammary epithelial cells derived from MMTV-*neu*mice revealed that HER2/NEU-driven changes in protein expression are functionally clustered. IUBMB Life.

[22] Lee GY, Park K, Kim SY, Byun Y (2007). MMPs-specific PEGylated peptide–DOX conjugate micelles that can contain free doxorubicin. Eur J Pharm Biopharm.

[23] Hondermarck H, Vercoutter-Edouart AS, Revillion F, Lemoine J, El-Yazidi-Belkoura I, Nurcombe V, Peyrat JP (2001). Proteomics of breast cancer for marker discovery and signal pathway profiling. Proteomics.

[24] Kaplan-Meier Plotter Breast Cancer. [http://kmplot.com/breast]

[25] Mongiat M, Fu J, Oldershaw R, Greenhalgh R, Gown AM, Iozzo RV (2003). Perlecan protein core interacts with extracellular matrix protein 1 (ECM1), a glycoprotein involved in bone formation and angiogenesis. J Biol Chem.

[26] Li Z, Zhang Y, Liu Z, Wu X, Zheng Y, Tao Z, Mao K, Wang J, Lin G, Tian L, Ji Y, Qin M, Sun S, Zhu X, Sun B (2011). ECM1 controls T_H_2 cell egress from lymph nodes through re-expression of S1P_1_. Nat Immunol.

[27] Merlin J, Stechly L, de Beauce S, Monte D, Leteurtre E, van Seuningen I, Huet G, Pigny P (2011). Galectin-3 regulates MUC1 and EGFR cellular distribution and EGFR downstream pathways in pancreatic cancer cells. Oncogene.

[28] Zhao Q, Guo X, Nash GB, Stone PC, Hilkens J, Rhodes JM, Yu LG (2009). Circulating galectin-3 promotes metastasis by modifying MUC1 localization on cancer cell surface. Cancer Res.

[29] Schroeder JA, Thompson MC, Gardner MM, Gendler SJ (2001). Transgenic MUC1 interacts with epidermal growth factor receptor and correlates with mitogen-activated protein kinase activation in the mouse mammary gland. J Biol Chem.

[30] Pochampalli MR, el Bejjani RM, Schroeder JA (2007). MUC1 is a novel regulator of ErbB1 receptor trafficking. Oncogene.

[31] Ochieng J, Fridman R, Nangia-Makker P, Kleiner DE, Liotta LA, Stetler-Stevenson WG, Raz A (1994). Galectin-3 is a novel substrate for human matrix metalloproteinases-2 and −9. Biochemistry.

[32] Nangia-Makker P, Raz T, Tait L, Hogan V, Fridman R, Raz A (2007). Galectin-3 cleavage: a novel surrogate marker for matrix metalloproteinase activity in growing breast cancers. Cancer Res.

[33] Sato H, Seiki M (1993). Regulatory mechanism of 92 kDa type IV collagenase gene expression which is associated with invasiveness of tumor cells. Oncogene.

[34] Savorè C, Zhang C, Muir C, Liu R, Wyrwa J, Shu J, Zhau HE, Chung LW, Carson DD, Farach-Carson MC (2005). Perlecan knockdown in metastatic prostate cancer cells reduces heparin-binding growth factor responses *in vitro* and tumor growth *in vivo*. Clin Exp Metastasis.

[35] Radke S, Austermann J, Russo-Marie F, Gerke V, Rescher U (2004). Specific association of annexin 1 with plasma membrane-resident and internalized EGF receptors mediated through the protein core domain. FEBS Lett.

[36] Hu Y, Gao H, Vo C, Ke C, Pan F, Yu L, Siegel E, Hess KR, Linskey ME, Zhou YH (2014). Anti-EGFR function of EFEMP1 in glioma cells and patient prognosis. Oncoscience.

[37] Sercu S, Zhang M, Oyama N, Hansen U, Ghalbzouri AE, Jun G, Geentjens K, Zhang L, Merregaert JH (2008). Interaction of extracellular matrix protein 1 with extracellular matrix components: ECM1 is a basement membrane protein of the skin. J Invest Dermatol.

[38] Garrett JT, Olivares MG, Rinehart C, Granja-Ingram ND, Sánchez V, Chakrabarty A, Dave B, Cook RS, Pao W, McKinely E, Manning HC, Chang J, Arteaga CL (2011). Transcriptional and posttranslational up-regulation of HER3 (ErbB3) compensates for inhibition of the HER2 tyrosine kinase. Proc Natl Acad Sci U S A.

[39] Liu W, Hsu DK, Chen HY, Yang RY, Carraway KL, Isseroff RR, Liu FT (2012). Galectin-3 regulates intracellular trafficking of EGFR through Alix and promotes keratinocyte migration. J Invest Dermatol.

[40] Hisatsune A, Nakayama H, Kawasaki M, Horie I, Miyata T, Isohama Y, Kim KC, Katsuki H (2011). Anti-MUC1 antibody inhibits EGF receptor signaling in cancer cells. Biochem Biophys Res Commun.

[41] Raina D, Uchida Y, Kharbanda A, Rajabi H, Panchamoorthy G, Jin C, Kharbanda S, Scaltriti M, Baselga J, Kufe D (2014). Targeting the MUC1-C oncoprotein downregulates HER2 activation and abrogates trastuzumab resistance in breast cancer cells. Oncogene.

[42] Iurisci I, Tinari N, Natoli C, Angelucci D, Cianchetti E, Iacobelli S (2000). Concentrations of galectin-3 in the sera of normal controls and cancer patients. Clin Cancer Res.

[43] Iorio MV, Casalini P, Piovan C, Di Leva G, Merlo A, Triulzi T, Ménard S, Croce CM (2009). Tagliabue E: **microRNA-205 regulates HER3 in human breast cancer**. Cancer Res.

[44] Nangia-Makker P, Wang Y, Raz T, Tait L, Balan V, Hogan V, Raz A (2010). Cleavage of galectin-3 by matrix metalloproteases induces angiogenesis in breast cancer. Int J Cancer.

[45] Fujimoto N, Terlizzi J, Aho S, Brittingham R, Fertala A, Oyama N, McGrath JA, Uitto J (2006). Extracellular matrix protein 1 inhibits the activity of matrix metalloproteinase 9 through high-affinity protein/protein interactions. Exp Dermatol.

[46] Xiong GP, Zhang JX, Gu SP, Wu YB, Liu JF (2012). Overexpression of ECM1 contributes to migration and invasion in cholangiocarcinoma cell. Neoplasma.

[47] Chattopadhyay S, Shubayev VI (2009). MMP-9 controls Schwann cell proliferation and phenotypic remodeling via IGF-1 and ErbB receptor-mediated activation of MEK/ERK pathway. Glia.

[48] Burns DM, He C, Li Y, Scherle P, Liu X, Marando CA, Covington MB, Yang G, Pan M, Turner S, Fridman JS, Hollis G, Vaddi K, Yeleswaram S, Newton R, Friedman S, Metcalf B, Yao W (2008). Conversion of an MMP-potent scaffold to an MMP-selective HER-2 sheddase inhibitor via scaffold hybridization and subtle P^′^_1_permutations. Bioorg Med Chem Lett.

[49] Colomer R, Montero S, Lluch A, Ojeda B, Barnadas A, Casado A, Massuti B, Cortés-Funes H, Lloveras B (2000). Circulating HER2 extracellular domain and resistance to chemotherapy in advanced breast cancer. Clin Cancer Res.

[50] Huang L, Chen D, Liu D, Yin L, Kharbanda S, Kufe D (2005). MUC1 oncoprotein blocks glycogen synthase kinase 3β-mediated phosphorylation and degradation of β-catenin. Cancer Res.

[51] Cascio S, Zhang L, Finn OJ (2011). MUC1 protein expression in tumor cells regulates transcription of proinflammatory cytokines by forming a complex with nuclear factor-κB p65 and binding to cytokine promoters: importance of extracellular domain. J Biol Chem.

[52] Rajabi H, Alam M, Takahashi H, Kharbanda A, Guha M, Ahmad R, Kufe D (2014). MUC1-C oncoprotein activates the ZEB1/miR-200c regulatory loop and epithelial–mesenchymal transition. Oncogene.

[53] Gu M, Guan J, Zhao L, Ni K, Li X, Han Z (2013). Correlation of ECM1 expression level with the pathogenesis and metastasis of laryngeal carcinoma. Int J Exp Pathol.

