# Correction to: ECM1 regulates cell proliferation and trastuzumab resistance through activation of EGF-signaling

**DOI:** 10.1186/s13058-019-1106-3

**Published:** 2019-03-26

**Authors:** Kyung-min Lee, Keesoo Nam, Sunhwa Oh, Juyeon Lim, Young-Pil Kim, Jong Won Lee, Jong-Han Yu, Sei-Hyun Ahn, Sung-Bae Kim, Dong-Young Noh, Taehoon Lee, Incheol Shin

**Affiliations:** 1 Department of Life Science, Hanyang University, Seoul, KS013 Republic of Korea; 2 Department of Surgery, College of Medicine, University of Ulsan and Asan Medical Center, Seoul, KS013 Republic of Korea; 3 Department of Oncology, College of Medicine, University of Ulsan and Asan Medical Center, Seoul, KS013 Republic of Korea; 4 Cancer Research Institute, Seoul National University College of Medicine, Seoul, KS013 Korea; 5 NOVA Cell Technology, Inc., Pohang, KS010 Republic of Korea; 6 Natural Science Institute, Hanyang University, Seoul, KS013 Republic of Korea

**Correction to: Breast Cancer Res**

**https://doi.org/10.1186/s13058-014-0479-6**

After the publication of this work [1] errors were found in Figs. 1a and 5d. In the second column of Fig. 1a, an image from matrigel cultures of BT-474 wild type cells were mistakenly used for BT-474 TR shE cells. In Fig. 5d, the same blots were inserted for immunoprecipiation: HER3/Western blots: Mucin1 and immunoprecipitation Mucin1/Western blots: HER3. The corrected figures are shown below. We confirmed that the corrected figures do not affect conclusion and findings of the article. We sincerely apologize for the errors.

**Fig. 1:**
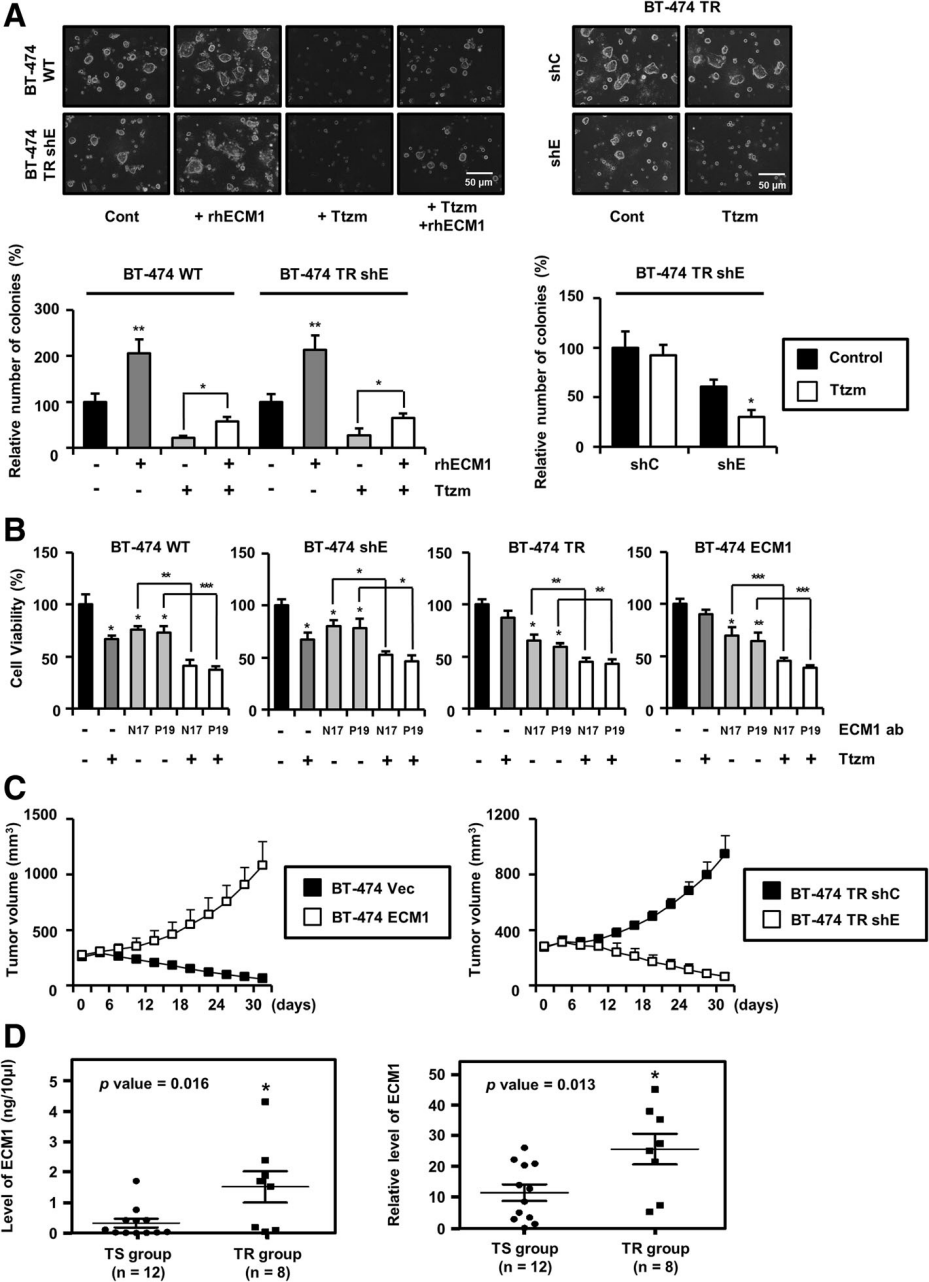
ECM1 confers resistance toward Ttzm. **a** Cells were seeded with matrigel and treated with Ttzm (20 μg/mL) and rhECM1 (200 ng/mL). The numbers of colonies 20 μm or greater in diameter were counted at 12 days (*p < 0.05, **p < 0.005). **b** At 24 h after cell seeding, each cell line was treated with anti-ECM1 antibody (5 μg/mL) and Ttzm (20 μg/mL) in fresh medium. After a further 48 h, cell viability was analyzed using an MTT assay (*p < 0.05, **p < 0.005, ***p < 0.0005). **c** BT-474 vector and ECM1 cells and BT-474 TR control shRNA and ECM1 shRNA cells were passaged in the mice by subcutaneous injection into the lower flank of each mouse. When the tumor size increased up to 250 mm^3^, Ttzm at 20 mg/kg was administered to each mouse by i.p. injection twice per week (*n* = 5 or 6 for each group). **d** Circulating levels of ECM1 in serum from Ttzm-resistant breast cancer patients were assessed by ELISA (left) and Western blot analysis, and compared with (right) corresponding data for Ttzm-responsive patients (*p < 0.05)

**Fig. 5:**
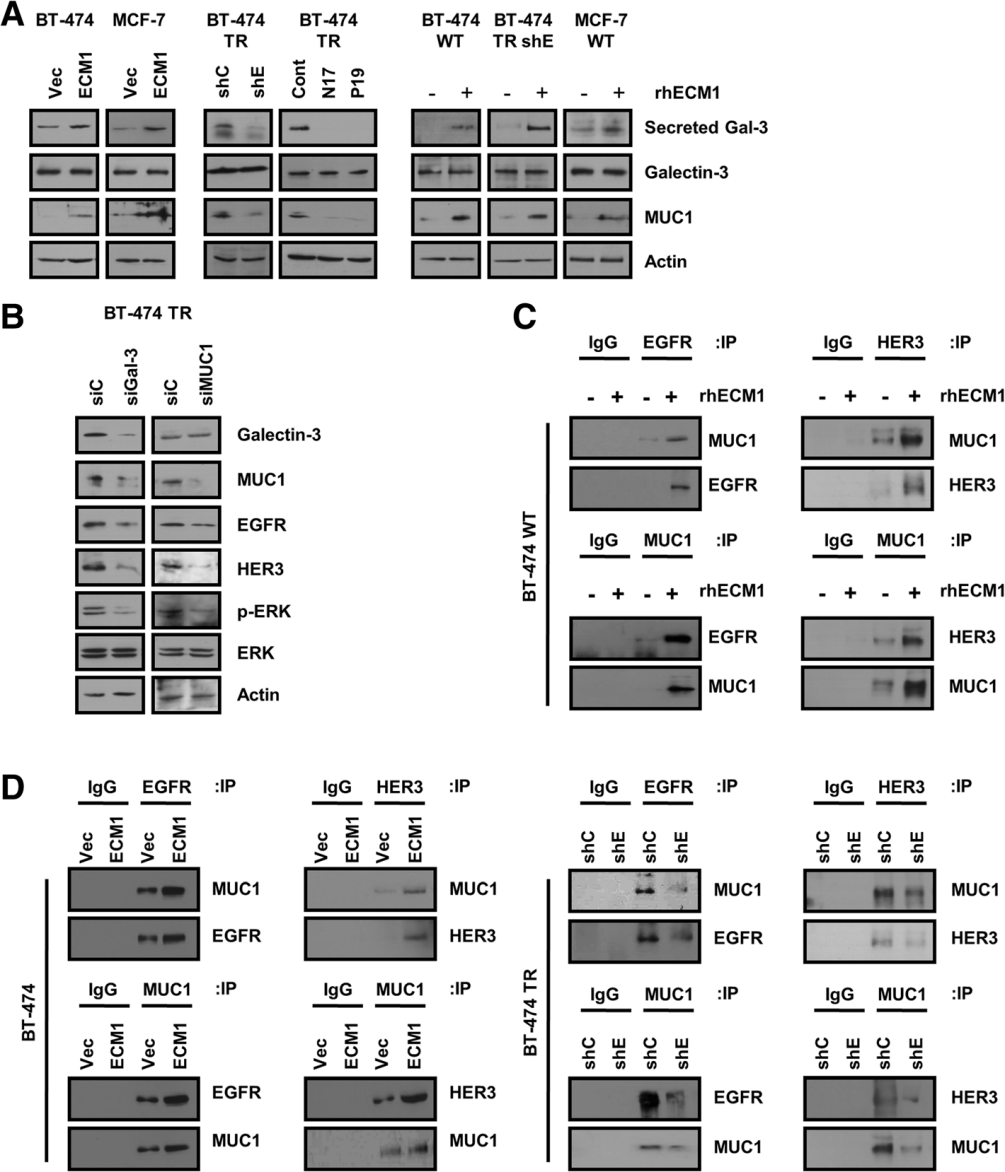
ECM1 stabilizes EGFR and HER3 proteins through galectin-3/MUC1. **a** Lysates from each cell line were analyzed by Western blotting. **b** At 24 h after seeding, BT-474 TR cells were transfected with each siRNA, incubated further for 48 h, and analyzed on Western blots. **c** At 24 h after seeding, cells were treated with rhECM1 (200 ng/mL) and incubated further for 48 h. Cell lysates were then incubated with MUC1, EGFR and HER3 antibodies overnight. Immunoprecipitates were analyzed on Western blots. **d** Total cell lysates were incubated with MUC1, EGFR and HER3 antibodies overnight, and immunoprecipitates were then analyzed on Western blots
