# Supplementary material for: Extracellular matrix protein 1 regulates cell proliferation and trastuzumab resistance through activation of epidermal growth factor signaling
Source: Breast Cancer Res. 2014 Dec 11;16:479. doi: 10.1186/s13058-014-0479-6 (PMC4308848; doi:10.1186/s13058-014-0479-6)
Supplement: Supplementary file 1 — Additional file 1: Table S1.: List of primers used in the study. Supplementary materials and methods. (PDF 71 KB) [file 13058_2014_479_MOESM1_ESM.pdf]

## Additional_file_1

### Table S1. List of primers used in the study

| Gene name | Seqence (5' - 3') | |
|---|---|---|
| | Fwd | Rev |
| ECM1 | CCC ACC CAT CTG AAC ACT CAT TAC | CAG GGC ACC AAG ATC CAC TTA AA |
| EGFR | ATA GTC GCC CAA AGT TCC GTG AGT | ACC ACG TCG TCC ATG TCT TCT TCA |
| HER2 | GCT GAA CAA TAC CAC CCC TGT CAC AG | TGT GAG AGC CAG CTG GTT CTT G |
| HER3 | AAT GGC TCG GGC TCT GAT ACT TGT | TAC TTG TAG ATT GGG CCC TTG GCA |
| MUC1 | CTG CTC CTC ACA GTG CTT ACA GTT G | TGA ACC GGG GCT GTG GCT GG |
| MMP-9 | ACT GCT GGC CCT TCT ACG GC | CTC CCC CTG CCC TCA GAG AA |
| GAPDH | TCG ACA GTC AGC CGC ATC TTC TTT | ACC AAA TCC GTT GAC TCC GAC CTT |

## Supplementary materials and methods

### Three-dimensional growth assays

Growth factor-reduced matrigel (BD Biosciences), diluted 1:3 with DMEM and containing 5 x $10^4$ cells/ml, was poured into 6-well plates, which were then incubated at 37ºC in 5% CO2 and 95% humidified air. The medium was replaced with DMEM containing trastuzumab every 3 days and colonies were photographed after 12 days. Cell colonies measuring over 20 µm were counted using a biological microscope equipped with OLYMPUS cellSens Standard 1.5 software.

### Soft agar colony forming assays

Colony forming assay using low-melting point agarose were done as following description in the presence of trastuzumab. Bottom layers containing DMEM with 10% FBS were consist of 0.8% of low-melting point agarose (Sigma-Aldrich) and 10 mM HEPES. Each bottom layers were poured on 6-well plates and solidified for 30 min at room temperature. Fifty thousand cells were seeded in top layers containing DMEM with 10% FBS, 0.4% low-melting point agarose and 5 mM HEPES. Colonies were photographed after 12 days.

### Reverse transcriptation-polymerase chain reaction (RT-PCR)

Expression of transcripts was assessed using the following primers, human ECM: Fwd 5’-AGG CTC GGT TCT CCT GCT TCC AG-3’, Rev 5’-TTG GGG TAA GGA GCC CGA CGG-3’ and human GAPDH primers: Fwd 5’-GGT GAA GGT CGG AGT CAA CG-3’, Rev 5’-CAA AGT TGT CAT GGA TGA CC-3’.

### Measure of miRNA expression

Total RNA was isolated from cells using the mirVana MicroRNA isolation kit (Lifetechnologies). cDNAs were prepared from 2 μg total RNA using the TaqMan MicroRNA Reverse transcription kit (Lifetechnologies). Expression of miR-200c was assessed by RT–qPCR with a universal reverse primer and forward primers specific for miR-200c using the TaqMan MicroRNA Assays kit (Life technologies).

**Zymography**

Zymography was performed on 10% polyacrylamide gels that containing 0.5 mg/mL gelatin (Sigma-Aldrich). Samples were resuspended in a zymography sample buffer without denaturation and run on a gel containing gelatin. After electrophoresis, the gels were incubated for 30 min at room temperature in a renaturing buffer (50 mM Tris; pH 7.4, 2% Triton X-100). After renaturation, the gels were incubated for 48 h at 37°C in developing buffer (50 mM Tris; pH 8.0, 5 mM $CaCl_2$, 0.02% $NaN_3$). After developing, the gels were stained with 0.1% Coomassie Blue R-250 for 30 min at room temperature and then the gels were destained with destaining solution (25% ethanol, 10% acetic acid).
