# Supplementary figures and images for: Extracellular matrix protein 1 regulates cell proliferation and trastuzumab resistance through activation of epidermal growth factor signaling
Source: Breast Cancer Res. 2014 Dec 11;16:479. doi: 10.1186/s13058-014-0479-6 (PMC4308848; doi:10.1186/s13058-014-0479-6)

Figure S1

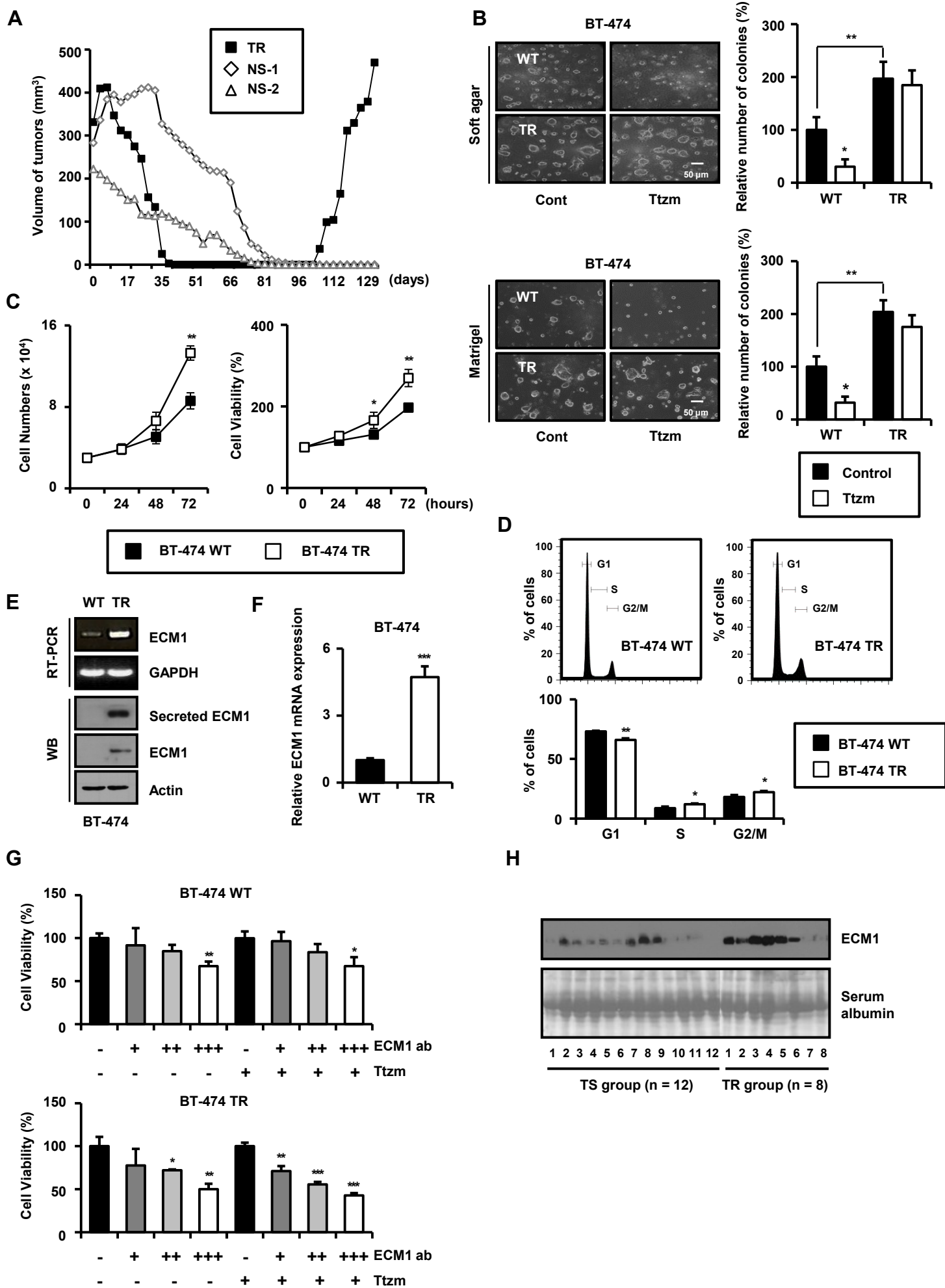

Supplement: Supplementary file 2 — Additional file 2: Figure S1.: ECM1 is over-expressed in Ttzm-resistant BT-474 cells. (A) 17β-estradiol pellet was injected to 4-week-old BABL/c nude mice and BT-474 WT cells were passaged in the mice by subcutaneous injection of 2 × 10^7^ cells into the lower flank of the mice. When the tumor size grew to 250 mm3, 20 mg/kg Ttzm was injected into the mice by intraperitoneal injection twice per week. NS-1 and NS-2, as control groups, responded to Ttzm completely. (B) 5 × 10^4^ cells were plated on soft agar and Matrigel (Additional file 1: Supplementary materials and methods). In Matrigel, Ttzm (20 μg/ml) was treated every 3 days. The number of colonies (20 μm diameter) was counted at 12 days. The number of colonies is quantified in right panels. Error bars represent mean ± SD of triplicate experiments (*P < 0.05, **P < 0.005). (C) Cells were counted with a hemocytometer over 3 days (*P < 0.05, **P < 0.005). (D) Cell cycles in BT-474 WT and BT-474 TR cells were analyzed using flow cytometry (*P < 0.05, **P < 0.005). (E) mRNAs were analyzed by RT-PCR using primers specific for ECM1 and GAPDH (Additional file 1: Supplementary materials and methods). Secreted ECM1 was obtained from Trichloroacetic acid-precipitated cell supernatant medium. Each cell lysate was analyzed by Western blotting using ECM1- and actin-specific antibodies. (F) ECM1 mRNA levels were determined by real-time PCR using primers specific for ECM1 (***P < 0.0005). (G) At 24 hours after cell seeding, each cell line was treated with anti-ECM1 antibody (5 μg/ml) and Ttzm (20 μg/ml) in fresh medium. After a further 48 hours, cell viability was analyzed using an MTT assay (*P < 0.05, **P < 0.005, ***P < 0.0005). (H) Levels of ECM1 in serum from Ttzm-resistant breast cancer patients were assessed Western blot analysis, and compared with corresponding data for Ttzm-responsive patients. (PDF 313 KB) [file 13058_2014_479_MOESM2_ESM.pdf]

Figure S2

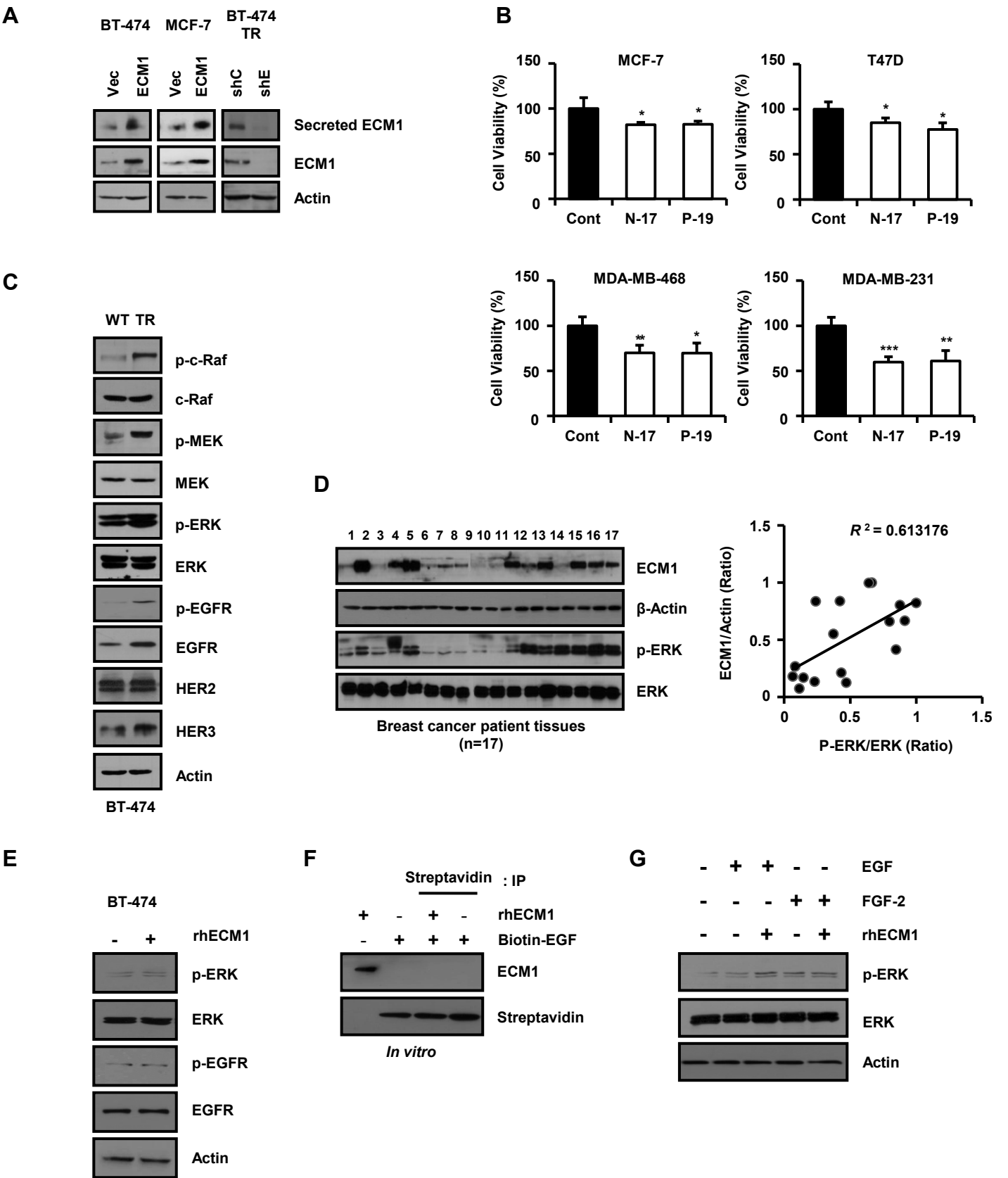

Supplement: Supplementary file 3 — Additional file 3: Figure S2.: Functional role of ECM1 in cancer cell proliferation. (A) Cells lysates were analyzed by Western blotting with the indicated antibodies. (B) Each cell line was treated with each anti-ECM1 antibody (see Methods) at 5 μg/ml. After a further 48 hours, cell viability was analyzed using an MTT assay (*P < 0.05, **P < 0.005, ***P < 0.0005). (C) Cell lysates were analyzed by Western blotting using indicated antibodies. Anti-actin antibody was applied as a loading control. (D) Western blot analysis shows levels of p-ERK and ECM1 proteins in primary tumor lysates from breast cancer patients (n = 17). The positive relationship between p-ERK and ECM1 expression levels is indicated (R 2 = 0.6131). (E) After serum starvation for 24 hours, cells were treated with rhECM1 (200 ng/ml) for 10 minutes. Cell lysates were analyzed by Western blotting using indicated antibodies. (F) A mixture containing rhECM1 (500 ng) and biotin-EGF (500 ng) was incubated with streptavidin-agarose beads overnight and the immunoprecipitates were analyzed on Western blots. (G) At 24 hours after cell seeding, BT-474 WT cells were treated with rhECM1 (200 ng/ml) and FGF2 (10 ng/ml). The cell lysates were obtained at the indicated time points and subjected to Western blot analysis with indicated antibodies. (PDF 353 KB) [file 13058_2014_479_MOESM3_ESM.pdf]

Figure S3

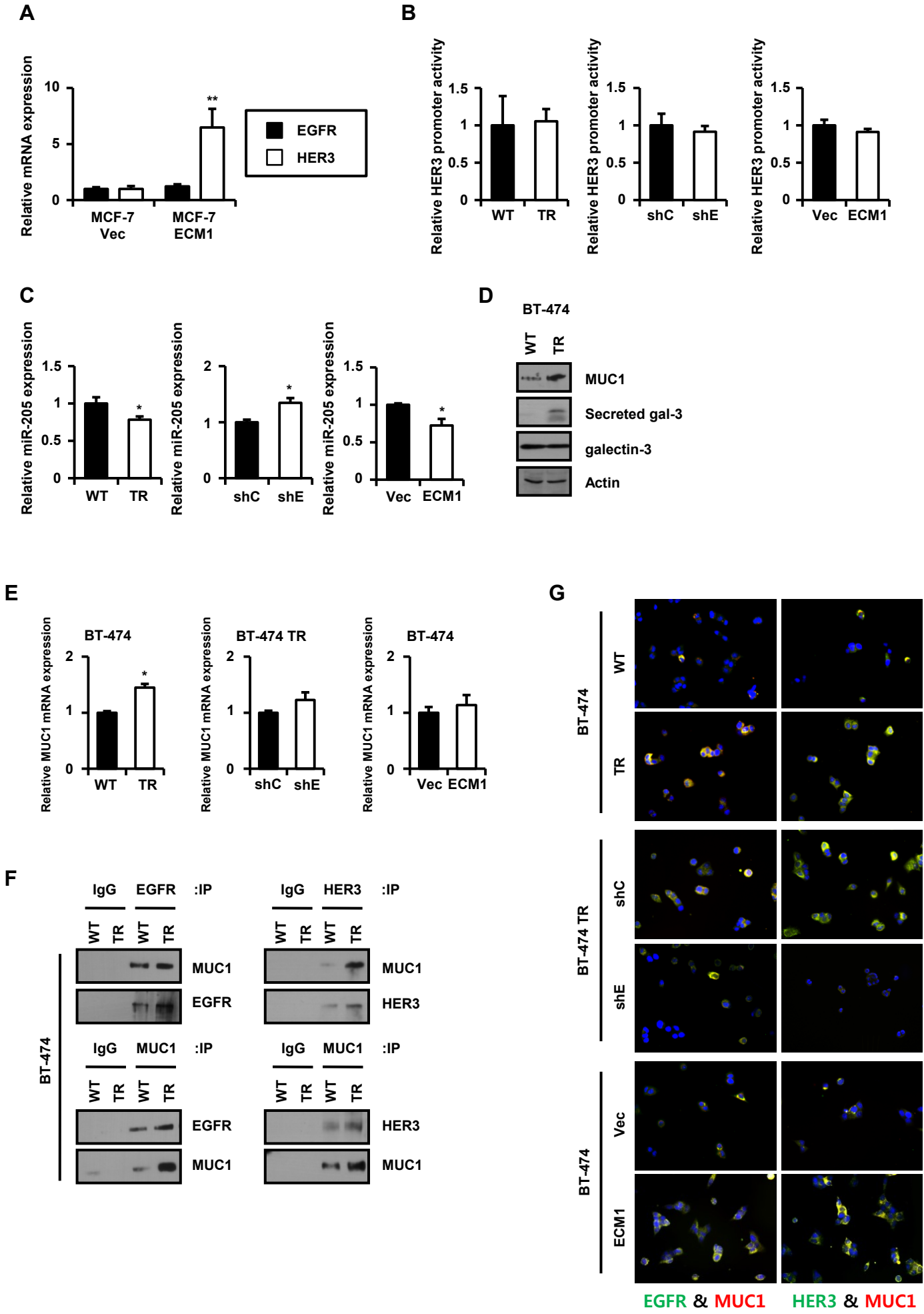

Supplement: Supplementary file 4 — Additional file 4: Figure S3.: ECM1-dependent induction of EGFR/HER3 is mediated by MUC1. (A) EGFR and HER3 mRNA levels were determined by real-time PCR using primers specific for EGFR and HER3 (**P < 0.005). (B) Each cell was transfected with HER3 promoter luciferase reporter constructs, harvested after 48 h and analyzed by dual-luciferase assay. (C) Expression of miR-200c was assessed by RT-qPCR with a universal reverse primer and forward primers specific for miR-200c using a TaqMan microRNA assay kit (*P < 0.05) (Additional file 1: Supplementary materials and methods). (D) Cell lysates were analyzed by Western blotting using the indicated antibodies. (E) MUC1 mRNA levels were determined by real-time PCR using primers specific for MUC1 (*P < 0.05). (F) Cell lysates were incubated with MUC1, EGFR and HER3 antibodies overnight. Immunoprecipitates were analyzed on Western blots. (G) Colocalizations of MUC1 and EGFR/HER3 were monitored by immunostaining. Each cell was fixed and stained with indicated antibodies and Hoechst dye for nuclear staining. (PDF 294 KB) [file 13058_2014_479_MOESM4_ESM.pdf]

Figure S4

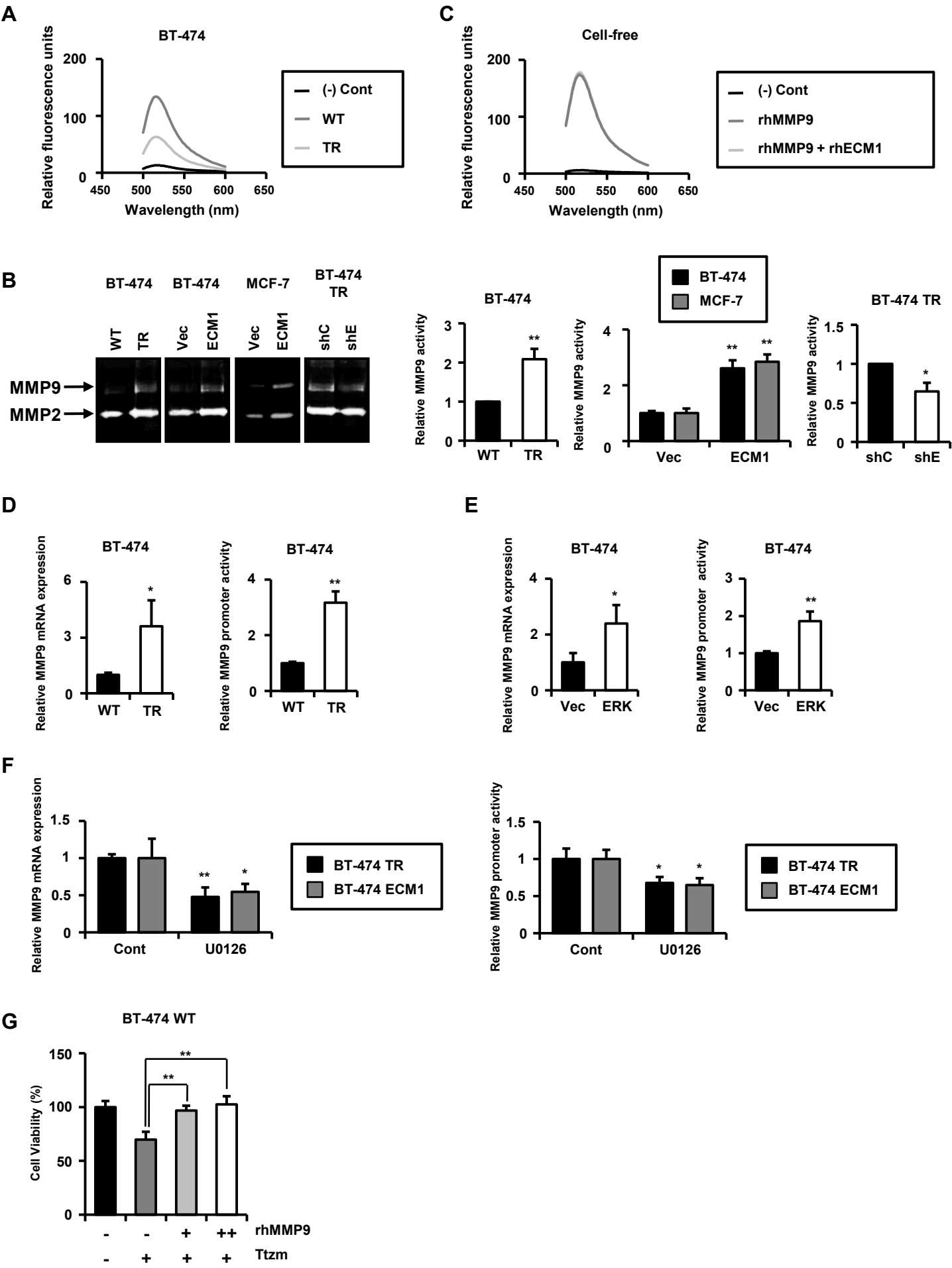

Supplement: Supplementary file 5 — Additional file 5: Figure S4.: ERK-dependent regulation of MMP9 transcription by ECM1. (A) Supernatant medium from each cell line was reacted with MMP9 substrate and relative fluorescence units were determined at 480 to 620 nm. (B) Conditioned media from each cell were collected, and gelatin zymography was performed. Arrows indicate MMP2 and MMP9. Each bar graph represents the quantified intensity of indicated cells, as assessed by gelatin zymography (*P < 0.05, **P < 0.005) (Additional file 1: Supplementary materials and methods). (C) Media containing rhMMP9 and rhECM1 were reacted with MMP9 substrate. Relative fluorescence units were determined at 480 to 620 nm. (D) MMP9 mRNA levels were determined by real-time PCR using primers specific for MMP9 (*P < 0.05). Each cell line was transfected with an MMP9 promoter luciferase reporter construct. After 48 h, cells were harvested and the lysates were analyzed by dual-luciferase assay (**P < 0.005). (E) and (F) Each cell was transfected with ERK1-WT constructs (E) and treated with U0126 (F). MMP9 mRNA levels were determined by real-time PCR using primers specific for MMP9 and MMP9 promoter activity was analyzed by dual-luciferase assay (*P < 0.05, **P < 0.005). (G) At 24 hours after cell seeding, each cell line was treated with rhMMP9 (10, 20 ng/ml) and Ttzm (20 μg/ml) and incubated further for 48 hours. Cell viability was then analyzed using an MTT assay (**P < 0.005). (PDF 150 KB) [file 13058_2014_479_MOESM5_ESM.pdf]

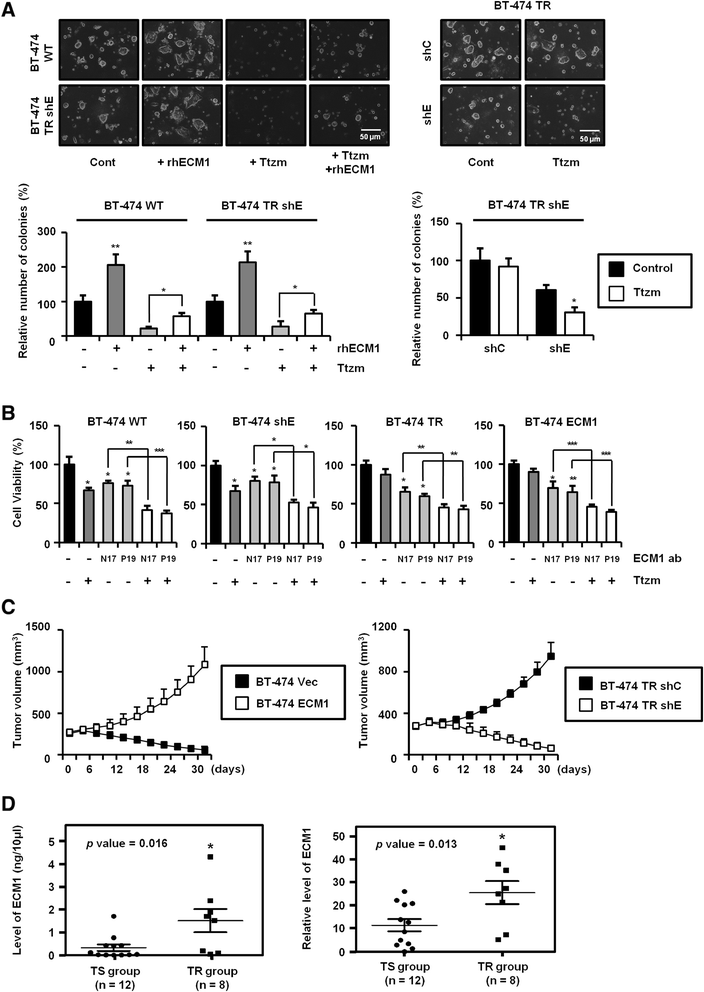

Supplement: Supplementary file 6 — Authors’ original file for figure 1 [file 13058_2014_479_MOESM6_ESM.gif]

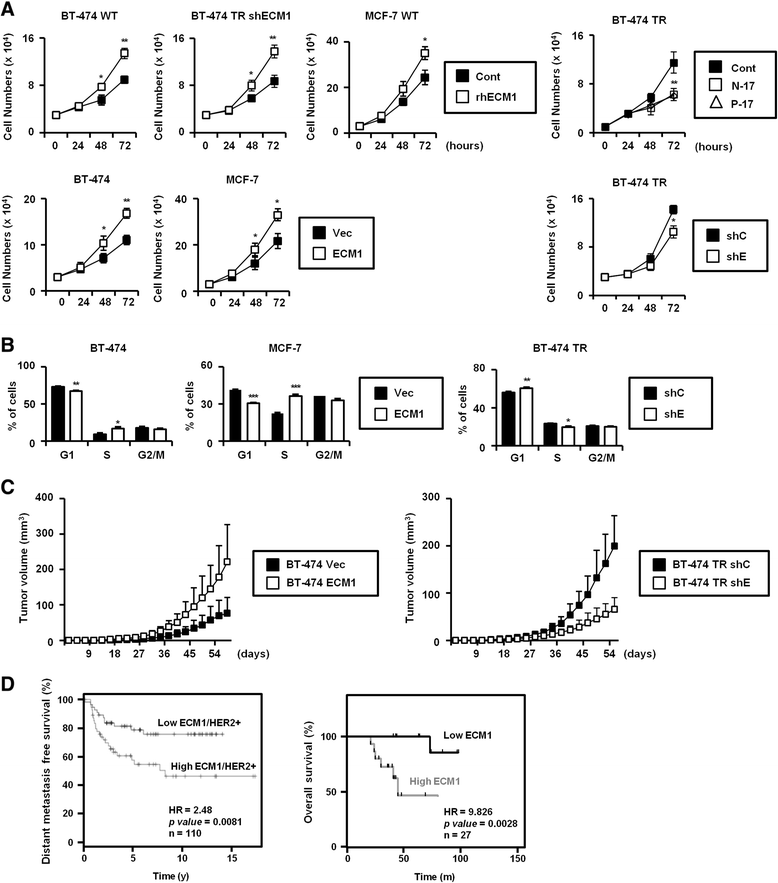

Supplement: Supplementary file 7 — Authors’ original file for figure 2 [file 13058_2014_479_MOESM7_ESM.gif]

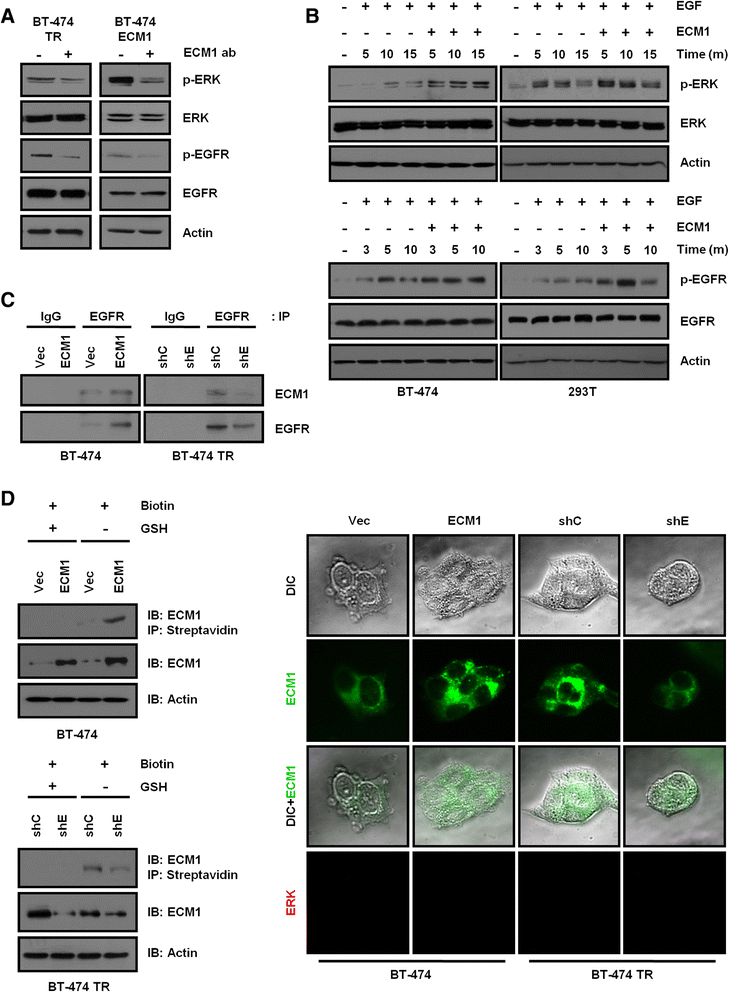

Supplement: Supplementary file 8 — Authors’ original file for figure 3 [file 13058_2014_479_MOESM8_ESM.gif]

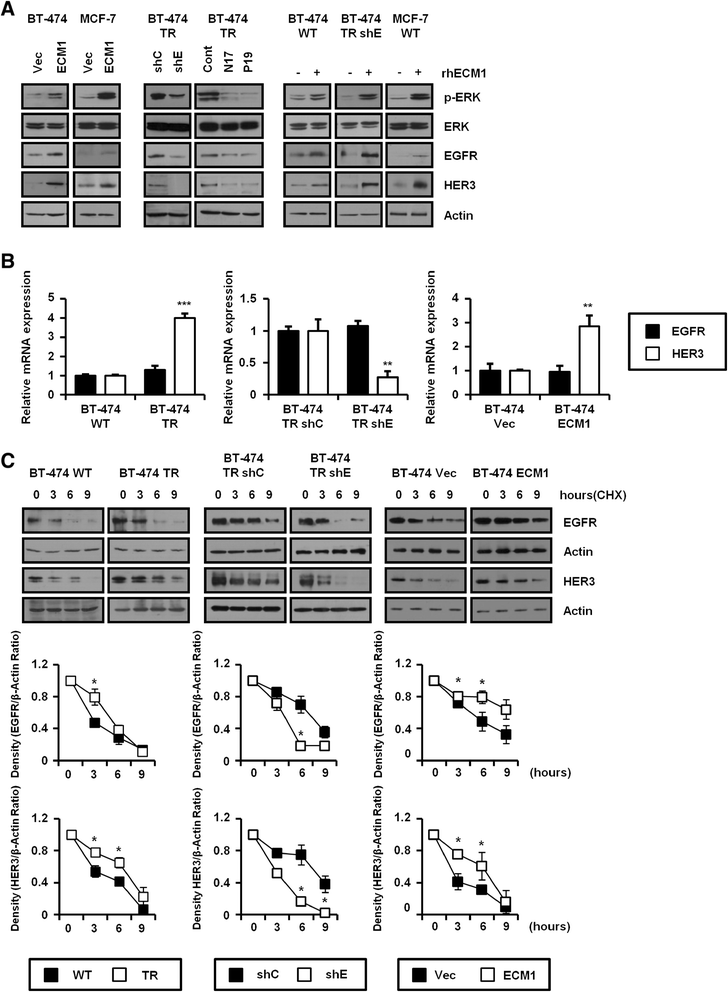

Supplement: Supplementary file 9 — Authors’ original file for figure 4 [file 13058_2014_479_MOESM9_ESM.gif]

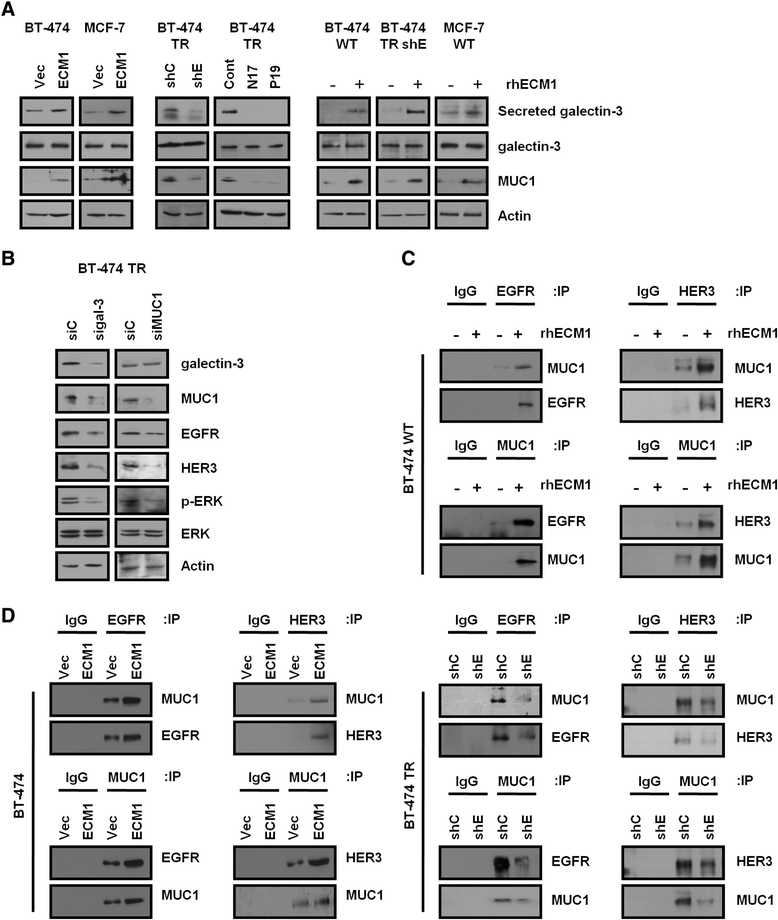

Supplement: Supplementary file 10 — Authors’ original file for figure 5 [file 13058_2014_479_MOESM10_ESM.gif]

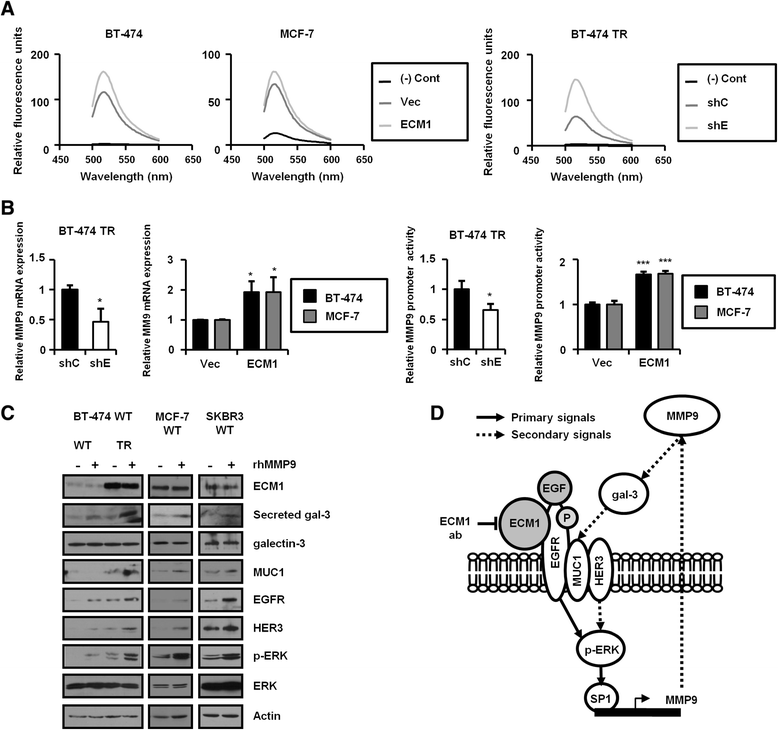

Supplement: Supplementary file 11 — Authors’ original file for figure 6 [file 13058_2014_479_MOESM11_ESM.gif]
